# Perception of structurally distinct effectors by the integrated WRKY domain of a plant immune receptor

**DOI:** 10.1073/pnas.2113996118

**Published:** 2021-12-08

**Authors:** Nitika Mukhi, Hannah Brown, Danylo Gorenkin, Pingtao Ding, Adam R. Bentham, Clare E. M. Stevenson, Jonathan D. G. Jones, Mark J. Banfield

**Affiliations:** ^a^Department of Biochemistry and Metabolism, John Innes Centre, Norwich NR4 7UH, United Kingdom;; ^b^The Sainsbury Laboratory, University of East Anglia, Norwich NR4 7UH, United Kingdom

**Keywords:** disease resistance, effector proteins, virulence, protein structure, plant biology

## Abstract

Plants use intracellular nucleotide-binding domain (NBD) and leucine-rich repeat (LRR)–containing immune receptors (NLRs) to detect pathogen-derived effector proteins. The *Arabidopsis* NLR pair RRS1-R/RPS4 confers disease resistance to different bacterial pathogens by perceiving the structurally distinct effectors AvrRps4 from *Pseudomonas syringae* pv. *pisi* and PopP2 from *Ralstonia solanacearum* via an integrated WRKY domain in RRS1-R. How the WRKY domain of RRS1 (RRS1^WRKY^) perceives distinct classes of effector to initiate an immune response is unknown. Here, we report the crystal structure of the in planta processed C-terminal domain of AvrRps4 (AvrRps4^C^) in complex with RRS1^WRKY^. Perception of AvrRps4^C^ by RRS1^WRKY^ is mediated by the β2-β3 segment of RRS1^WRKY^ that binds an electronegative patch on the surface of AvrRps4^C^. Structure-based mutations that disrupt AvrRps4^C^–RRS1^WRKY^ interactions in vitro compromise RRS1/RPS4-dependent immune responses. We also show that AvrRps4^C^ can associate with the WRKY domain of the related but distinct RRS1B/RPS4B NLR pair, and the DNA-binding domain of *At*WRKY41, with similar binding affinities and how effector binding interferes with WRKY–W-box DNA interactions. This work demonstrates how integrated domains in plant NLRs can directly bind structurally distinct effectors to initiate immunity.

## Significance

This study reveals a mechanism for effector perception by a plant NLR immune receptor that contains an integrated domain (ID) that mimics an authentic effector target. The *Arabidopsis* immune receptors RRS1 and RPS4 detect the *Pseudomonas syringae* pv. *pisi* secreted effector AvrRps4 via a WRKY ID in RRS1. We used structural biology to reveal the mechanisms of AvrRps4^C^–WRKY interaction and demonstrated that this binding is essential for effector recognition in planta. Our analysis revealed features of the WRKY ID that mediate perception of structurally distinct effectors from different bacterial pathogens. These insights could enable engineering NLRs with novel recognition specificities, and enhance our understanding of how effectors interact with host proteins to promote virulence.

Plants coevolve with their pathogens, resulting in extensive genetic variation in host immune receptor and pathogen virulence factor (effector) repertoires (1). To enable host colonization, pathogenic microbes deliver effector proteins into host cells that suppress host immune responses and elevate host susceptibility by manipulating host physiology (2, 3). Plants have evolved surveillance mechanisms to detect and then activate defenses that combat pathogens, and detect host-translocated effectors via nucleotide-binding domain (NBD) and leucine-rich repeat (LRR)–containing receptors (NLRs) (4). NLR genes are highly diverse, showing both copy-number and presence/absence of polymorphisms, and different alleles can exhibit distinct effector recognition specificities (5, 6). As described by the gene-for-gene model, plant NLRs usually recognize a single effector (7). However, NLRs capable of responding to multiple effectors are known (5, 8, 9).

NLRs typically contain an N-terminal Toll/interleukin-1 receptor (TIR) or coiled-coil (CC) domain, a central NBD (NB-ARC [NBD shared with APAF-1, various R proteins, and CED-4]), and a C-terminal LRR domain (6). In addition to these canonical domains, some NLRs have evolved to carry integrated domains that mimic effector virulence targets and facilitate immune activation by directly binding effectors (10–15). Interestingly, integrated domain-containing NLRs (NLR-IDs) usually function with a paired helper NLR, which is required for immune signaling (10, 16).

The *Arabidopsis* NLR pair RRS1-R/RPS4 is a particularly interesting NLR-ID/NLR pair that confers resistance to bacterial pathogens *Pseudomonas syringae* and *Ralstonia solanacearum*, and also to a fungal pathogen (*Colletotrichum higginsianum*) where the effector is unknown (17–20). RRS1-R contains an integrated WRKY domain near its C terminus (RRS1^WRKY^), which interacts with two structurally distinct type III secreted bacterial effectors, AvrRps4 from *P. syringae* pv. *pisi* and PopP2 from *R. solanacearum* (13, 14, 21, 22). The RRS1^WRKY^ domain may mimic the DNA-binding domain of WRKY transcription factors (TFs), the putative virulence targets of AvrRps4 and PopP2, to enable immune perception of these effectors (13). Two alleles of RRS1 have been identified that differ in the length of the C-terminal extension after the WRKY domain (*SI Appendix*, Fig. S1). RRS1-R, from the accession Ws-2, has a 101–amino acid C-terminal extension beyond the end of the WRKY domain, and can perceive AvrRps4 and PopP2, while RRS1-S from Col-0, which perceives AvrRps4 but not PopP2, is likely a derived allele with a premature stop codon, and has only an 18–amino acid C-terminal extension (23). Most *Arabidopsis* ecotypes also carry a paralogous and genetically linked RRS1B/RPS4B NLR pair, which only perceives AvrRps4 (24). RRS1B/RPS4B share a similar domain architecture with RRS1/RPS4, including 60% sequence identity in the integrated WRKY domain.

AvrRps4 is proteolytically processed in planta to produce a 133–amino acid N-terminal fragment (AvrRps4^N^) and an 88–amino acid C-terminal fragment (AvrRps4^C^) (25, 26). Previous studies have highlighted the role of AvrRps4^C^ in triggering RRS1/RPS4-dependent immune responses (25, 26). AvrRps4^N^ has been reported to potentiate immune signaling from AvrRps4^C^ (27, 28). PopP2 is sequence and structurally distinct from AvrRps4 and has an acetyltransferase activity that is likely related to its role in virulence. The structural basis of PopP2 perception by RRS1^WRKY^ has been determined (29), but how RRS1^WRKY^ binds AvrRps4^C^ and whether this is via a shared or different interface to PopP2 is unknown.

Here, we determined the structural basis of AvrRps4^C^ recognition by the integrated WRKY ID of RRS1. The recognition of AvrRps4^C^ is mediated by the β2-β3 segment of RRS1^WRKY^, the same region used to bind PopP2. This segment interacts with surface-exposed acidic residues of AvrRps4^C^. Structure-informed mutagenesis at the AvrRps4^C^–RRS1^WRKY^ interface identifies AvrRps4 residues required for protein–protein interactions in vitro and in planta and AvrRps4 perception and immune responses. Residues mediating the interaction of AvrRps4^C^ and RRS1^WRKY^ are conserved in both the RRS1B^WRKY^ and the DNA-binding domain of WRKY TFs, and AvrRps4^C^ mutants that prevent interaction with RRS1^WRKY^ also disrupt binding to *At*WRKY41. This supports the hypothesis that the RRS1^WRKY^ mimics host WRKY TFs through a shared effector-binding mechanism. We also show that AvrRps4^C^ prevents the interaction of RRS1^WRKY^ and *At*WRKY41 with W-box DNA, most likely via steric blocking, at the same WRKY domain site acetylated by PopP2.

## Results

### AvrRps4^C^ Interacts with the Integrated WRKY Domain of RRS1 In Vitro.

**Fig. 1.**
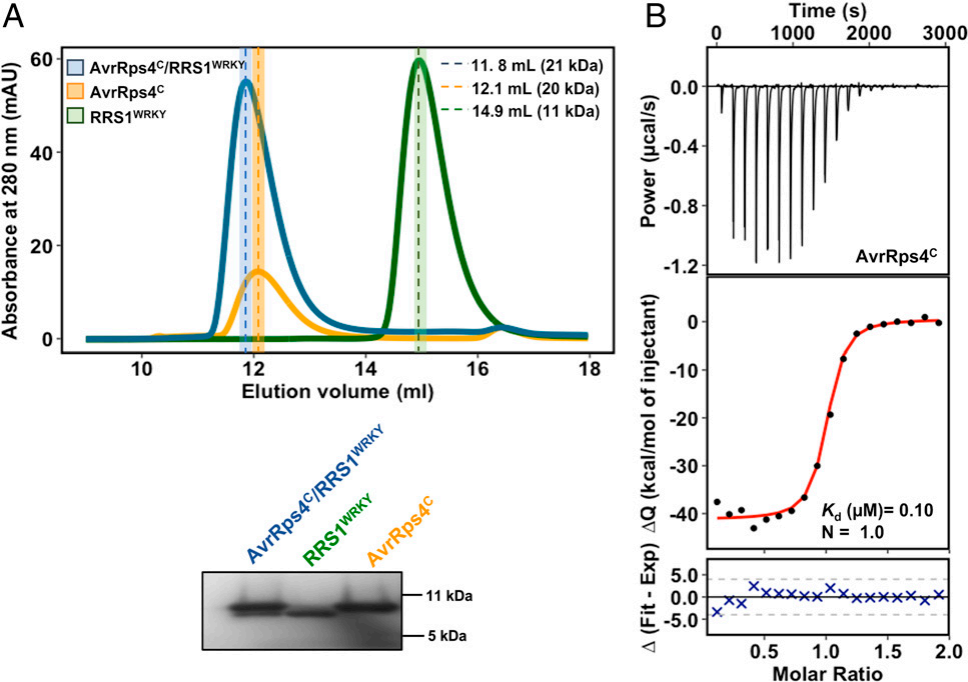
AvrRps4^C^ interacts with the WRKY domain of RRS1 in vitro. (*A*) Analytical gel filtration traces (using a Superdex 75 10/300 column) for AvrRps4^C^ alone (gold), RRS1^WRKY^ alone (green), and AvrRps4^C^ with RRS1^WRKY^ (blue) with sodium dodecyl sulfate–polyacrylamide gels of relevant fractions. An equimolar ratio of AvrRps4^C^ and RRS1^WRKY^ was used for the analysis. AvrRps4^C^ runs as a dimer in vitro. Poor absorbance for AvrRps4^C^ at 280 nm is due to its low molar extinction coefficient. (*B*) ITC titrations of AvrRps4^C^ with RRS1^WRKY^. (*B*, *Upper*) Raw processed thermogram after baseline correction and noise removal. (*B*, *Lower*) The experimental binding isotherm obtained for the interaction of AvrRps4^C^ and RRS1^WRKY^ together with the global fitted curves (displayed in red) were obtained from three independent experiments using AFFINImeter software (61). *K*_d_ and binding stoichiometry (*N*) were derived from fitting to a 1:1 binding model.

To investigate how AvrRps4^C^ interacts with the RRS1^WRKY^ domain, constructs comprising residues 134 to 221 of AvrRps4^C^ (the in planta processed C-terminal fragment) and residues 1194 to 1273 of RRS1-R (corresponding to the RRS1^WRKY^ domain) were separately expressed in *Escherichia coli* and proteins were purified via a combination of immobilized metal-affinity chromatography (IMAC) via 6×His tags and gel filtration (Superdex 75 26/60 and Superdex S75 16/60) (see *SI Appendix*, *Materials and Methods* for full details). We qualitatively assessed the interaction of purified AvrRps4^C^ with RRS1^WRKY^ using analytical gel filtration chromatography. Individually, the proteins displayed well-separated elution profiles. RRS1^WRKY^ eluted at a volume (*V*_e_) of 14.9 mL and AvrRps4^C^ eluted at a *V*_e_ of 12.1 mL (Fig. 1*A*). Following incubation of a 1:1 molar ratio of the proteins, we observed a new elution peak with an earlier *V*_e_ of 11.8 mL, and a lack of absorption peaks for the separate proteins (Fig. 1*A*). This demonstrates complex formation in vitro and suggests a 1:1 stoichiometry of the AvrRps4^C^–RRS1^WRKY^ complex.

We then determined the binding affinities of the interaction using isothermal titration calorimetry (ITC). Titration of AvrRps4^C^ into a solution of RRS1^WRKY^ resulted in an exothermic binding isotherm with a fitted dissociation equilibrium constant (*K*_d_) of 0.103 μM (Fig. 1*B*) and stoichiometry of 1:1. The thermodynamic parameters of the interaction are given in *SI Appendix*, Table S1. As RRS1^WRKY^ may be a mimic of WRKY TFs, we explored the binding kinetics of AvrRps4^C^ with *At*WRKY41 and *At*WRKY70 by ITC [previous reports have shown that AvrRps4 interacts with these proteins in yeast two-hybrid assay and by in planta coimmunoprecipitation (13, 30)]. We chose *At*WRKY41 for further study as this protein expressed and purified stably from *E. coli*. AvrRps4^C^ interacted with *At*WRKY41 with a *K*_d_ of 0.02 μM, and with similar thermodynamic parameters as RRS1^WRKY^ (*SI Appendix*, Fig. S2 and Table S1).

### Crystal Structure of the AvrRps4^C^–RRS1^WRKY^ Complex.

To reveal the molecular basis of the AvrRps4^C^ and RRS1^WRKY^ interaction, we coexpressed the proteins in *E. coli*, purified the complex, and obtained crystals that diffracted with 2.65-Å resolution at the Diamond Light Source (*SI Appendix*, *Materials and Methods*). The crystal structure of the AvrRps4^C^–RRS1^WRKY^ complex was solved by molecular replacement using the structure of RRS1^WRKY^ (from the PopP2–RRS1^WRKY^ complex, Protein Data Bank [PDB] ID code 5W3X) and AvrRps4^C^ (PDB ID code 4B6X) as models (*SI Appendix*, *Materials and Methods*). X-ray data collection, refinement, and validation statistics are shown in *SI Appendix*, Table S2.

**Fig. 2.**
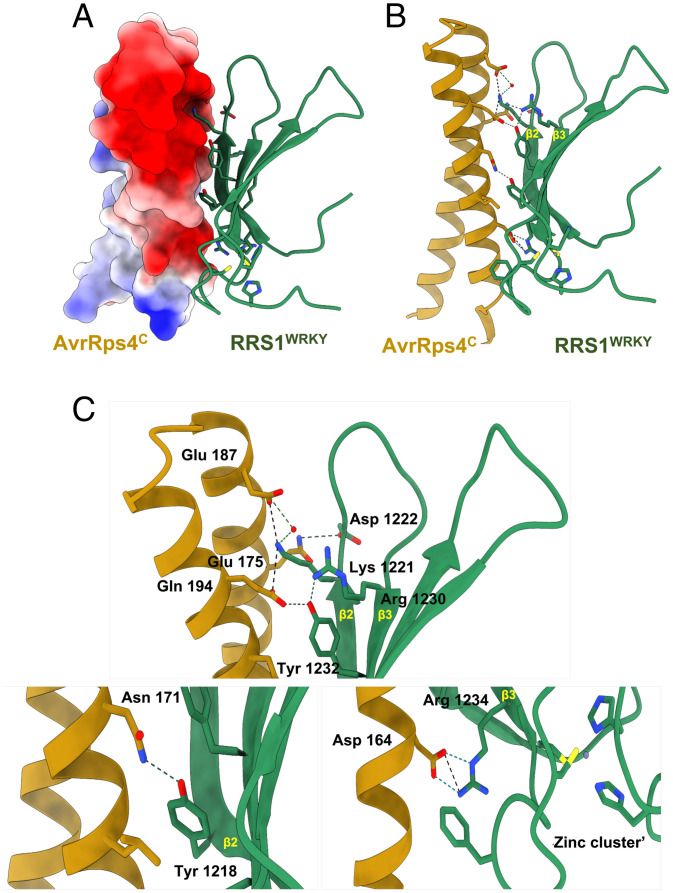
Structure of the AvrRps4^C^–RRS1^WRKY^ complex. (*A*) Electrostatic surface representation of AvrRps4^C^ in the AvrRps4^C^–RRS1^WRKY^ crystal structure displaying a prominent negative patch in AvrRps4 at the interacting interface. (*B*) Schematic representation of AvrRps4^C^–RRS1^WRKY^, highlighting interfacing residues. AvrRps4^C^ is shown in gold cartoon and RRS1^WRKY^ is shown in green with surface-exposed side chains as sticks. (*C*) Close-up view of the interactions of AvrRps4^C^ with the β2-β3 segment of RRS1^WRKY^. Hydrogen bonds are shown as dashed lines, and water molecules are depicted as red spheres. The Zn^2+^ ion is also displayed.

The structure comprises a 1:1 complex of AvrRps4^C^ and RRS1^WRKY^ (Fig. 2*A*), which supports the 1:1 binding model in ITC. Overall, AvrRps4^C^ adopts the same antiparallel α-helical CC structure in both free [PDB ID code 4B6X (26)] and complexed forms, with an rmsd of 0.66 Å over 59 C_α_ atoms (*SI Appendix*, Fig. S3*A*). Also, RRS1^WRKY^ adopts a conventional WRKY domain fold [rmsd of 2.03 Å over 61 C_α_ atoms compared with *At*WRKY1, PDB ID code 2AYD (31)] comprising a four-stranded antiparallel β-sheet (β2 to β5) stabilized by a zinc ion (C_2_H_2_ type). Comparison of RRS1^WRKY^ in the AvrRps4^C^–RRS1^WRKY^ and PopP2–RRS1^WRKY^ complex (PDB ID code 5W3X) structures reveals high conformational similarity, with an rmsd of 1.81 Å over 64 C_α_ atoms. The characteristic WRKY sequence signature motif WRKYGQK maps to the β2-strand of RRS1^WRKY^ and is directly involved in contacting AvrRps4^C^ (Fig. 2*B* and *SI Appendix*, Figs. S3*A* and S4). The same surface, including the β2-β3 strands of RRS1^WRKY^, forms contacts with PopP2 in the PopP2–RRS1^WRKY^ complex (29) (*SI Appendix*, Fig. S4), and mutants at this surface showed it to be essential for PopP2 recognition.

### The AvrRps4^C^–RRS1^WRKY^–Binding Interface Is Dominated by Electrostatic and Polar Interactions.

The total interface area buried in the AvrRps4^C^–RRS1^WRKY^ complex is 591.8 Å^2^, encompassing 12.3% (589.7 Å^2^) and 11.9% (593.9 Å^2^) of the total accessible surface areas of the effector and integrated domain, respectively [as calculated by PDBePISA (32); full details are given in *SI Appendix*, Table S3]. The binding interface between AvrRps4^C^ and RRS1^WRKY^ is largely formed by residues from the β2-β3 strand of RRS1^WRKY^, which present a positive surface patch that interacts with acidic residues on the surface of AvrRps4^C^ (Fig. 2*A*, *SI Appendix*, Fig. S3*B*, and Movie S1). The interaction between the β2-segment of RRS1^WRKY^, which harbors the WRKYGQK motif, and AvrRps4^C^ includes hydrogen bonds and/or salt-bridge interactions involving Tyr1218 and Lys1221 of RRS1^WRKY^ and AvrRps4 Glu175, Glu187, and Asn171. Notably, the side chain of RRS1^WRKY^ Lys1221 protrudes into an acidic cleft on the surface of AvrRps4^C^ to contact the side chains of both AvrRps4 Glu175 and Glu187 (Fig. 2 *B* and *C* and Movie S1). The OH atom of RRS1^WRKY^ Tyr1218 forms a hydrogen bond with the ND2 atom of AvrRps4 Asn171 (Fig. 2 *B* and *C*). Additional intermolecular contacts are formed by the β2-β3 loop of RRS1^WRKY^ involving the backbone carbonyl oxygen and nitrogen of Asp1222, which form hydrogen bonds with the side chains of AvrRps4 Asn190 and Gln194. The complex between AvrRps4^C^ and RRS1^WRKY^ is further stabilized by the β3-strand of RRS1^WRKY^ that forms hydrogen bonds and salt-bridge interactions via side chains of RRS1^WRKY^ Arg1230, Tyr1232, and Arg1234 to AvrRps4 Glu175 and Asp164 (Fig. 2 *B* and *C*). A detailed interaction summary is provided in *SI Appendix*, Table S4.

### Structure-Based Mutations in AvrRps4^C^ Perturb Binding to RRS1^WRKY^ In Vitro.

**Fig. 3.**
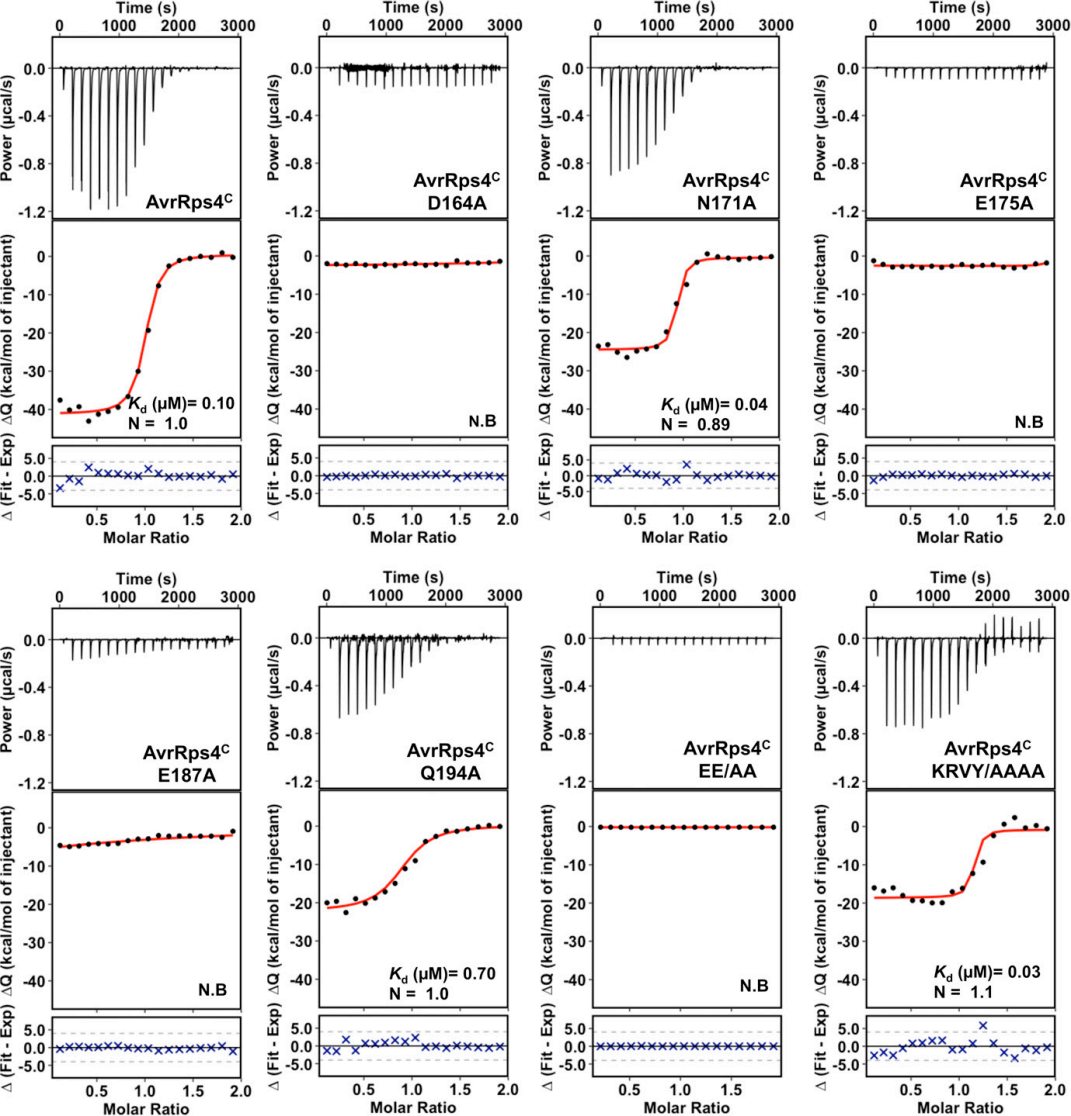
Structure-guided mutants of AvrRps4^C^ at the AvrRps4^C^–RRS1^WRKY^ interface disrupt interaction with RRS1^WRKY^ in vitro. ITC titrations of wild-type AvrRps4^C^ and mutants with RRS1^WRKY^. (*Upper*) Raw processed thermograms after baseline correction and noise removal. (*Lower*) Experimental binding isotherms obtained for the interaction of AvrRps4^C^ wild type and mutants with RRS1^WRKY^ together with the global fitted curves (displayed in red) obtained from three independent experiments using AFFINImeter software (61). *K*_d_ was derived from fitting to a 1:1 binding model. N.B., nonbinding.

To evaluate the contribution of residues at the AvrRps4^C^–RRS1^WRKY^ interface to complex formation in vitro, we generated six structure-guided mutants in AvrRps4^C^ (native amino acid to Ala) and tested the effect on protein interactions by ITC. Each AvrRps4^C^ mutant was purified from *E. coli* under the same conditions as for the wild-type protein, and proper folding was evaluated by circular dichroism (CD) spectroscopy (*SI Appendix*, Fig. S5). ITC titrations were carried out as for the wild-type interactions. Individual ITC isotherms are shown in Fig. 3, and the thermodynamic parameters of the interactions are shown in *SI Appendix*, Table S1. We found that mutating AvrRps4 residues Asp164 (D164A), Glu175 (E175A), Glu187 (E187A), and double mutant Glu175/Glu187 (EE/AA) essentially abolished complex formation in vitro (Fig. 3). Mutations in residues Asn171 (N171A) and Gln194 (Q194A) retained binding to RRS1^WRKY^, with N171A displaying wild-type levels and Q194A showing an approximately sevenfold reduction in affinity. Besides structure-guided mutants, we also tested binding of an AvrRps4 quadruple mutant, carrying mutations in the N-terminal KRVY motif (KRVY/AAAA) [previously identified to be essential for the virulence activity and perception of AvrRps4 (25)], with RRS1^WRKY^. Unlike most interface mutants, the AvrRps4^C^ KRVY/AAAA mutant retained wild type–like binding affinity with RRS1^WRKY^ (Fig. 3).

Since AvrRps4^C^ binds RRS1^WRKY^ and *At*WRKY41 with similar affinity (*SI Appendix*, Fig. S2), we tested the impact of the AvrRps4^C^ EE/AA double mutant on the binding to *At*WRKY41. We found that this mutant also abolishes interaction with *At*WRKY41, suggesting the same AvrRps4-binding interface is shared with different WRKY proteins (*SI Appendix*, Fig. S2).

### Structure-Based Mutations in AvrRps4 Prevent RRS1/RPS4-Mediated Cell Death in *Nicotiana tabacum*.

**Fig. 4.**
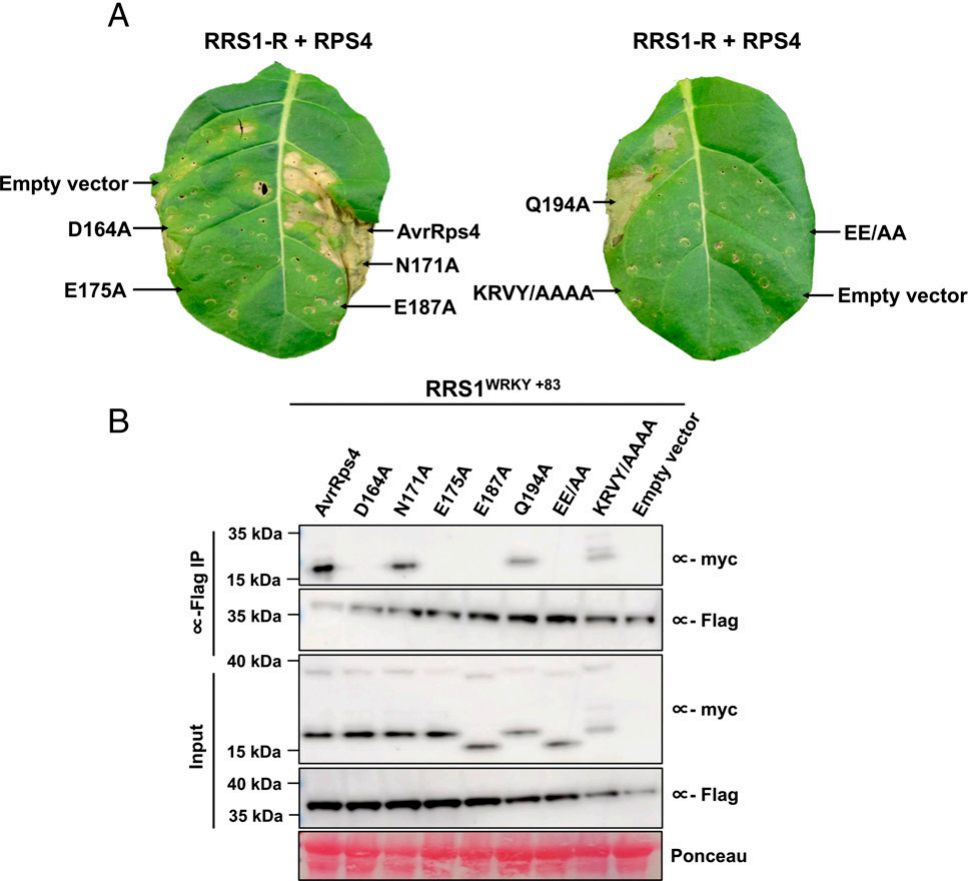
Structure-guided mutants of AvrRps4 at the AvrRps4^C^–RRS1^WRKY^ interface compromise RRS1-R/RPS4–mediated cell-death responses and in vivo binding in *Nicotiana*. (*A*) Representative leaf images showing RRS1-R/RPS4–mediated cell-death response to wild-type structure-guided mutants of AvrRps4. Agroinfiltration assays were performed in 4- to 5-wk-old *N. tabacum* leaves, and cell death was assessed at 4 d post infiltration. The experiment was repeated three times with similar results. (*B*) Coimmunoprecipitation (co-IP) of RRS1-R^WRKY+83^ (6×His/3×FLAG-tagged) with AvrRps4 and variants (4×myc-tagged) in *N. benthamiana*. Blots show protein accumulations in total protein extracts (input) and immunoprecipitates obtained with anti-FLAG magnetic beads when probed with appropriate antisera. Empty vector was used as a control. The experiment was repeated at least three times, with similar results.

To validate the biological relevance of the AvrRps4^C^–RRS1^WRKY^ interface observed in the crystal structure, we tested the effect of the AvrRps4^C^ mutants above on RRS1-R/RPS4–mediated immunity by monitoring the cell-death response in *N. tabacum*. *Agrobacterium*-mediated transient expression of wild-type AvrRps4 triggers a hypersensitive cell-death response (HR) 5 d post infiltration when coexpressed with RRS1-R/RPS4 (Fig. 4*A*). The previously characterized inactive AvrRps4 KRVY/AAAA mutant (25, 26) was used as a negative control. We found that AvrRps4 mutations at positions D164, E175, and E187 and the double mutant E175/E187 prevented RRS1-R/RPS4–dependent cell-death responses, consistent with their loss of binding to RRS1^WRKY^ in vitro (Fig. 4*A*). Interestingly, the N171A mutation, which retained its binding to RRS1^WRKY^ in vitro, displayed wild type–like cell death–inducing activity, and Q194A with an approximately sevenfold reduction in RRS1^WRKY^ affinity consistently exhibited a weaker cell-death response. Expression of all mutants was confirmed by immunoblotting (Fig. 4*B*). In addition to RRS1-R/RPS4, we also explored the effect of AvrRps4 structure-based mutations on RRS1-S/RPS4–dependent cell death in *N. tabacum* (*SI Appendix*, Figs. S1 and S6*A*). We found that AvrRps4 variants elicited similar immune responses when transiently coexpressed with RRS1-S/RPS4 or RRS1-R/RPS4.

### Loss of Cell Death in *N. tabacum* Correlates with the Loss of Binding to RRS1^WRKY^ In Vivo.

To determine whether loss of RRS1-R/RPS4–mediated HR in transient assays correlates with the loss of AvrRps4 binding to RRS1^WRKY^ in vivo as well as in vitro, we performed coimmunoprecipitation assays using full-length C-terminal 4×myc-tagged AvrRps4 constructs and C-terminal 6×His/3×FLAG-tagged constructs of RRS1-R^WRKY+83^ (equivalent to RRS1-D5/6R as defined in ref. 23). Wild-type AvrRps4 associates with RRS1-R^WRKY+83^ in its in planta processed form (Fig. 4*B*). Consistent with the cell-death phenotype and in vitro binding data, no association between AvrRps4 mutants D164A, E175A, E187A, or EE/AA and RRS1^WRKY+83^ was detected (Fig. 4*B*). Further, we observed wild-type levels of association of AvrRps4 N171A with RRS1^WRKY+83^, while AvrRps4 Q194A appeared to coimmunoprecipitate weakly. The AvrRps4 KRVY/AAAA mutant displayed wild type–like binding affinity toward RRS1^WRKY+83^, as observed previously (26).

### Structure-Guided Mutations in AvrRps4 Prevent HR in *Arabidopsis*.

**Fig. 5.**
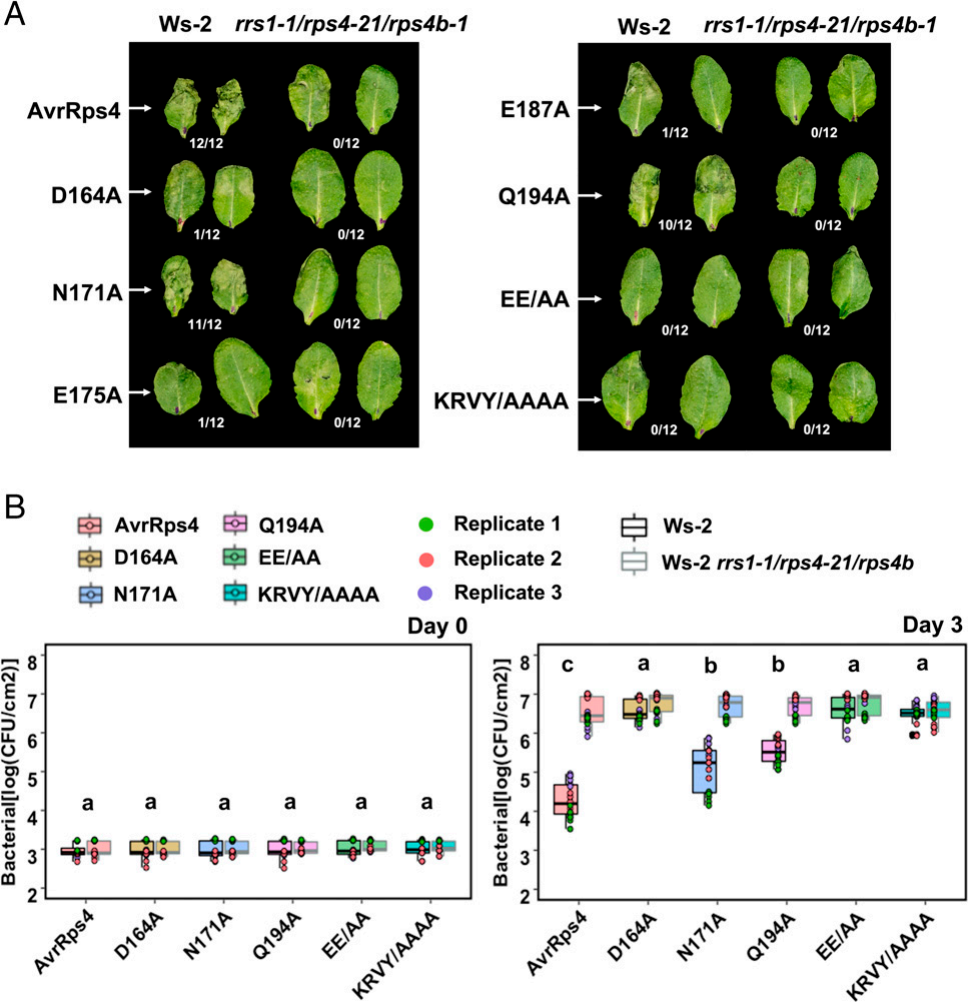
Structure-guided mutants of AvrRps4 compromise RRS1-R/RPS4–dependent recognition specificities and restriction of bacterial growth in *Arabidopsis*. (*A*) HR assay in different *Arabidopsis* accessions using *P. fluorescens* Pf0-1 secreting AvrRps4 wild type and structure-guided mutants. Constructs were delivered to the *Arabidopsis* Ws-2 and *rrs1-1/rps4-21/rps4b-1* knockout background and HR was recorded 20 h post infiltration. Fraction refers to the number of leaves showing HR of 12 randomly inoculated leaves. This experiment was repeated at least three times with similar results. (*B*) In planta bacterial growth assays of *Pto* DC3000 secreting AvrRps4 wild type and mutant constructs. Bacterial suspensions with OD_600_ = 0.001 were pressure-infiltrated into the leaves of 4- to 5-wk-old *Arabidopsis* plants. Values are plotted from three independent experiments (denoted in different colors). Statistical significance of the values was calculated by one-way ANOVA followed by post hoc Tukey honestly significant difference analysis. Letters above the data points denote significant differences (*P* < 0.05). A detailed statistical summary can be found in *SI Appendix*, Table S5. CFU, colony forming unit.

Next, we investigated the impact of AvrRps4 structure-guided mutations on the activation of RRS1-R/RPS4–dependent immune responses using HR assays in *Arabidopsis*. Constructs carrying full-length AvrRps4 wild type and mutants, flanked by a 126-bp native AvrRps4 promoter, were delivered into plant cells by infiltration using the Pf0-EtHAn (*Pseudomonas fluorescens* effector-to-host analyzer, hence Pf0) system (33). HR assays used *Arabidopsis* ecotype Ws-2 (encoding RRS1-R/RPS4 and RPS4B/RRS1B) and Ws-2 *rrs1-1/rps4-21/rps4b-1* (RRS1-R/RPS4/RPS4B triple-knockout) lines and were scored at 20 h post infiltration. Pf0 carrying wild-type AvrRps4 triggered HR in Ws-2, but not in Ws-2 *rrs1-1/rps4-21/rps4b-1*, as previously reported (13, 26). AvrRps4 KRVY/AAAA, an HR inactive mutant, was used as a negative control (26). The structure-guided mutants AvrRps4 D164A, E175A, E187A, and EE/AA all showed a complete loss of HR in Ws-2, with AvrRps4 Q194A showing a weaker HR and N171A showing a wild type–like phenotype (Fig. 5*A*). None of the AvrRps4 variants triggered HR in Ws-2 *rrs1-1/rps4-21/rps4b-1* (Fig. 5*A*).

In addition to Ws-2, we also performed a parallel set of experiments in *Arabidopsis* ecotype Col-0 (which encodes the RRS1-S allele) and the Col-0 *rrs1-3/rrs1b-1* (RRS1-S/RRS1B double-knockout) line. Overall, we observed a weaker HR toward AvrRps4 wild type and mutants in Col-0 in comparison with Ws-2. Nevertheless, a similar pattern of HR phenotypes was observed in Col-0 compared with Ws-2, and none of the AvrRps4 variants triggered HR in the Col-0 *rrs1-3/rrs1b-1* line (*SI Appendix*, Fig. S6*B*). The pattern of HR phenotypes conferred by the AvrRps4 interface mutants further validates the AvrRps4^C^–RRS1^WRKY^ structure and the role of these residues in recognition of AvrRps4 by the RRS1/RPS4 receptor pair.

### Loss of HR Correlates with Bacterial Growth in *Arabidopsis*.

Having demonstrated the role of AvrRps4 interface residues in effector-triggered HR in *Arabidopsis*, we next investigated their effects on bacterial growth. We performed bacterial growth assays on *Arabidopsis* ecotypes Ws-2, Col-0, Ws-2 *rrs1-1/rps4-21/rps4b-1*, and Col-0 *rrs1-3/rrs1b-1* using the *P. syringae* pv. *tomato* (*Pto*) DC3000 strain carrying AvrRps4 wild type or structure-based mutants. Since both the single mutants AvrRps4 E175A and E187A displayed the same impaired HR as the double AvrRps4 EE/AA mutant in previous assays, we focused on AvrRps4 EE/AA only for this assay. Bacterial growth was scored at 3 d post infection. *Pto* DC3000 carrying wild-type AvrRps4 displayed reduced growth on Ws-2 when compared with the mutant background (Ws-2 *rrs1-1/rps4-21/rps4b-1*), presumably due to the activation of RRS1-R/RPS4–dependent immunity (Fig. 5*B*). The effector mutants AvrRps4 D164A, EE/AA, and KRVY/AAAA, which displayed a complete loss of HR in Ws-2, showed a severe or complete lack of restriction of bacterial growth in Ws-2 (Fig. 5*B*). *Pto* DC3000:AvrRps4 Q194A and *Pto* DC3000:AvrRps4 N171A showed reduced bacterial growth (but not full restriction) when compared with wild-type AvrRps4, even though they displayed a similar cell-death phenotype in *N. tabacum* (albeit weaker for AvrRps4 Q194A) and HR in *Arabidopsis* (Figs. 4*A* and 5*A*). All the *Pto* DC3000:AvrRps4 variants tested displayed indistinguishable bacterial growth in the RRS1-R/RPS4 loss-of-function line (Fig. 5*B*). Finally, all the *Pto* DC3000:AvrRps4 variants displayed similar bacterial growth profiles in the Col-0 and Col-0 *rrs1-3/rrs1b-1* line when compared with Ws-2 and Ws-2 *rrs1-1/rps4-21/rps4b-1* (*SI Appendix*, Fig. S6*C*).

### The RRS1B/RPS4B Immune Receptor Pair Displays Similar Recognition Specificities toward AvrRps4 Variants as RRS1/RPS4.

In addition to RRS1/RPS4, the RRS1B/RPS4B pair can confer recognition of AvrRps4 in *Arabidopsis* (24). Sequence alignment revealed an overall 60% amino acid identity of the integrated WRKY domains from RRS1 and RRS1B, with the WRKYGQK motif and all residues interfacing with AvrRps4^C^ conserved (*SI Appendix*, Fig. S7). To explore AvrRps4 recognition by RRS1B/RPS4B, we performed ITC titrations of RRS1B^WRKY^ with wild-type AvrRps4^C^ in vitro. We found that RRS1B^WRKY^ binds to AvrRps4^C^ three times more weakly than RRS1^WRKY^ (*SI Appendix*, Fig. S7), possibly due to subtle changes imposed by residues outside the direct binding interface. When comparing the binding kinetics with the strength of immune responses in planta, we observed a weaker RRS1B/RPS4B-dependent HR to AvrRps4 compared with RRS1/RPS4. Nonetheless, both NLR pairs displayed a similar profile of immune responses toward the AvrRps4 structure-guided mutants in transient cell-death assays and in *Arabidopsis* HR assays (*SI Appendix*, Fig. S7).

### AvrRps4^C^ Interferes with WRKY–W-Box DNA Interactions.

**Fig. 6.**
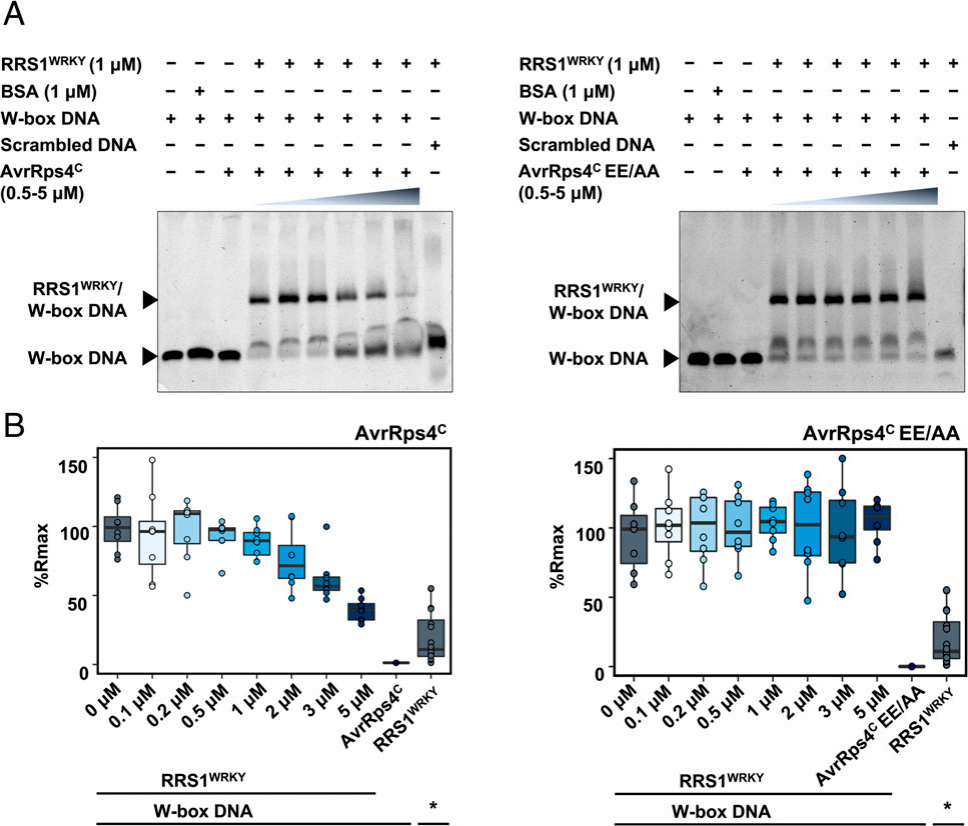
AvrRps4 interferes with W-box DNA binding by the RRS1^WRKY^ domain. (*A*) EMSA of DNA binding by RRS1^WRKY^ following preincubation of increasing concentrations of AvrRps4^C^ or AvrRps4^C^ EE/AA mutant. Bovine serum albumin (BSA) was used as a negative control for W-box DNA binding. Scrambled DNA was used as a negative control to test the specificity of RRS1^WRKY^ to W-box DNA. The experiment was repeated three times with similar results. (*B*) An SPR ReDCaT assay was performed using W-box and scrambled DNA (as a negative control). Percentage of normalized response (% *R*_max_) of RRS1^WRKY^ binding to W-box DNA and scrambled DNA (denoted by an asterisk) immobilized on a ReDCaT SPR chip. Titrations were performed following preincubation of 2 μM RRS1^WRKY^ with increasing concentrations of AvrRps4^C^ wild type and AvrRps4^C^ EE/AA mutant. The experiment was performed in eight replicates (each dot represents one replicate).

To regulate gene expression, WRKY TFs bind to specific W-box DNA motifs in the promoters of their target genes (34–36). Intriguingly, the majority of the AvrRps4-interacting residues are conserved within the DNA-binding domain of WRKY TFs (*SI Appendix*, Fig. S4 and Movie S2) and are indispensable for DNA binding (34). To test if AvrRps4 interferes with the W-box DNA-binding activity of RRS1^WRKY^ and *At*WRKY41, we preincubated increasing concentrations of AvrRps4^C^ and the AvrRps4^C^ EE/AA mutant (as a negative control) and studied their effect on the DNA-binding capacity of RRS1^WRKY^ and *At*WRKY41 using both electrophoretic mobility-shift assay (EMSA)– and surface plasmon resonance (SPR)–based assays. We found that the interaction of RRS1^WRKY^ and *At*WRKY41 with W-box DNA was reduced after preincubation with increasing concentrations of AvrRps4^C^ but not the AvrRps4^C^ EE/AA mutant (Fig. 6 and *SI Appendix*, Figs. S8–S10), revealing that AvrRps4^C^ interferes with WRKY binding to W-box DNA.

## Discussion

Despite recent advances, structural knowledge of how diverse integrated domains in plant NLRs perceive pathogen effectors is limited. Here, we investigated how the integrated WRKY domain of the *Arabidopsis* NLR RRS1 binds to the *Pseudomonas* effector AvrRps4, and how this underpins RRS1/RPS4-dependent immunity in planta. Further, through this work, we gained insights into interfaces in the RRS1^WRKY^ domain that are crucial for perception of two structurally unrelated effectors from distinct bacterial pathogens, which may have implications for NLR integrated domain engineering.

Transcriptional reprogramming upon NLR activation is well-established as an early immune response in plants (37–39), and direct interactions between NLRs and TFs have been reported (40–44). WRKY TFs are important molecular players in the regulation of plant growth and development and abiotic and biotic stresses (35, 36, 45). Typically, WRKY TFs target genes by binding W-box DNA in promoters, via a signature amino acid motif, WRKYGQK, to either promote or repress transcription (34, 46–48). As WRKY TFs play an important role in plant immunity, it is unsurprising that they are often found as integrated domains in NLR immune receptors (49), supporting the hypothesis that pathogen effectors enhance virulence by targeting WRKY TFs. Therefore, understanding how effectors bind to WRKY integrated domains may inform how effector/WRKY binding promotes disease. The structure of the AvrRps4^C^–RRS1^WRKY^ complex reveals that the effector directly interacts with the DNA-binding WRKYGQK motif, likely rendering it unavailable for binding to DNA (*SI Appendix*, Fig. S4 and Movie S2). AvrRps4^C^ binds to *At*WRKY41 with similar thermodynamic parameters to RRS1^WRKY^, and interface mutants that prevent AvrRps4^C^ interaction with RRS1^WRKY^ prevent interaction with *At*WRKY41, supporting the hypothesis that AvrRps4^C^ binds different WRKY proteins via a similar interface. WRKY TFs bind W-box DNA sequences in the promoters of their target genes. We used EMSAs and SPR assays to observe how the interaction of AvrRps4^C^ with RRS1^WRKY^ or *At*WRKY41 affects the binding of these proteins to a generic W-box DNA sequence. Preincubation of RRS1^WRKY^ and *At*WRKY41 with increasing amounts of AvrRps4^C^ reduced DNA-binding activity, whereas preincubation with AvrRps4 EE/AA showed no significant difference (Fig. 6 and *SI Appendix*, Figs. S8–S10). WRKY domain residues interacting with AvrRps4^C^ are well-conserved in these TFs (*SI Appendix*, Fig. S11), suggesting that AvrRps4 could interfere with and sterically block DNA binding of multiple WRKY TFs, thus promoting virulence. In addition to WRKY TFs, a recent publication suggests AvrRps4 can interact with BTS (nucleus-located Fe sensor BRUTUS) domains to affect pathogen colonization (50). Understanding whether these functions are related requires further investigation.

Comparing the AvrRps4^C^–RRS1^WRKY^ structure with that of PopP2–RRS1^WRKY^ (29) reveals an overlapping binding site for the effectors, primarily mediated by the β2-β3 segment of the WRKY domain (*SI Appendix*, Fig. S4 and Movie S2). The second lysine of the WRKYGQK motif, Lys1221, is acetylated by PopP2, abolishing the affinity of the WRKY domain for W-box DNA (13, 14, 29). Intriguingly, this acetylation event also abolished the association of AvrRps4^C^ with RRS1^WRKY^ (13), highlighting the important role of this interface in mediating the association of RRS1^WRKY^ with both effectors. It also highlights the likely shared role of these effectors in preventing interaction of WRKY domains with DNA as their virulence activity, either via enzymatic modification or steric blocking.

Studies with the NLR pair Pik from rice have shown that the strength of effector binding to integrated domains in vitro can correlate with immune responses in planta (51–53). Of the AvrRps4 mutants we tested to validate the RRS1^WRKY^ interface, all except N171A and Q194A prevented binding in vitro (by ITC) and in planta (by coimmunoprecipitation), and these did not give cell death in *Nicotiana* species when coexpressed with either RRS1-R/RPS4 or RRS1-S/RPS4. Further, they did not give HR or restrict bacterial growth in *Arabidopsis* Ws-2 or Col-0 ecotypes (except for a partial restriction of bacterial growth for the D164A mutation in the Col-0 background). The N171A mutant retained the same level of binding as wild type in vitro, and displayed the same in planta phenotypes, although restriction of bacterial growth in *Arabidopsis* was reduced compared with wild type in both Ws-2 and Col-0 ecotypes. Finally, the Q194A mutant showed a reduced binding in vitro (approximately sevenfold compared with wild type) but maintained an HR in *Arabidopsis* as well as displaying a restriction of bacterial growth in *Arabidopsis*, albeit reduced compared with wild type. Interestingly, this mutant consistently showed a qualitative reduction in the intensity of cell death in *Nicotiana*. Taken together, these AvrRps4 mutations validate the complex with RRS1^WRKY^ in that they prevent interaction in vitro and in planta, but they are not sufficient to determine whether strength of binding in vitro can directly correlate with in planta phenotypes. Further studies, including additional mutants, will be required to study this in the RRS1/RPS4 system.

Structural studies of singleton NLRs have shown that interactions between effectors and multiple domains within an NLR can be essential for activation (54–57). It is yet to be established whether this is also the case for effector perception involving paired NLRs with integrated domains, although the rice blast pathogen effector AVR-Pia immunoprecipitates with its sensor NLR Pia-2 (RGA5) when the integrated HMA domain has been deleted. However, this interaction does not promote immune responses in planta (58). Although unresolved in the structure of AvrRps4^C^ alone, or in complex with RRS1^WRKY^, the N-terminal KRVY motif is known to be required for both the virulence activity of the effector and its perception by RRS1/RPS4 (25, 26). Here, we verified that the quadruple mutant AvrRps4 KRVY/AAAA retains interaction with RRS1^WRKY^ at wild-type levels in vitro and in vivo, but did not trigger RRS1/RPS4-dependent responses in our in planta assays. This suggests that while binding of AvrRps4 to the RRS1^WRKY^ domain is essential for immune activation, an additional interaction mediated by the N-terminal region of the effector to a region of RRS1 and/or RPS4 outside this domain is also required for initiation of defense. Further studies are required to determine how additional receptor domains outside of integrated domains in NLR-IDs contribute to receptor function.

The *Arabidopsis* NLR pair RRS1B/RPS4B perceives AvrRps4, but not PopP2 (24). Phylogenetically, the RRS1 WRKY belongs to group III of the WRKY superfamily, whereas RRS1B^WRKY^ is grouped into group IIe (14, 24, 48). Here we found that AvrRps4^C^ binds RRS1B^WRKY^ with threefold lower affinity and RRS1B/RPS4B shows a similar pattern of recognition specificity in planta but with reduced phenotypes compared with RRS1/RPS4. A full investigation addressing why AvrRps4 shows differential interaction strength and phenotypes between RRS1 and RRS1B is beyond the scope of this work, but will be a direction for future research.

The unique ability of RRS1/RPS4 to perceive two effectors that differ both in sequence and structure, via the same integrated domain, highlights the potential for engineering of sensor NLRs to recognize diverse effectors. Recently, the range of rice blast pathogen effectors recognized by the integrated HMA domain of Pia-2 (RGA5) has been expanded by molecular engineering (58). However, this expanded recognition was toward structurally related effectors and may not be via a shared interface. Further, although cell-death responses were observed in *Nicotiana benthamiana*, the engineered NLR was not able to deliver an expanded disease resistance profile in transgenic rice. This suggests we still require a better understanding of how NLR-IDs interact with effectors, and their partner helper NLRs, to enable bespoke engineering of disease resistance.

## Materials and Methods

### Gene Cloning.

For in vitro studies, the gene fragments of AvrRps4^C^ (Gly134 to Gln221), RRS1^WRKY^ (Ser1194 to Thr1273), RRS1B^WRKY^ (Asn1164 to Thr1241), and *At*WRKY41 (Thr125 to Ile204) were cloned in various pOPIN expression vectors using an in-fusion cloning strategy as described in *SI Appendix*, *Materials and Methods*.

For transient assays in *N. tabacum* and *N. benthamiana*, domesticated genomic fragments encoding RRS1-R, RRS1-S, RRS1B, RPS4, and RPS4B were cloned into binary vector pICSL86977 under a 35S (CaMV) promoter with a C-terminal 6×His/3×FLAG tag using the Golden Gate assembly method as described (23). Similar cloning techniques were used to generate constructs expressing RRS1^WRKY+83^. Full-length AvrRps4 (*P. syringae* pv. *pisi*) was PCR-amplified from published constructs (13, 23, 26) and assembled with a C-terminal 4×myc tag in binary vector pICSL86977 under the control of the 35S (CaMV) promoter using the Golden Gate assembly method. DNA encoding each mutation was synthesized and cloned into pICSL86977 as described above.

For HR and bacterial growth assays in *Arabidopsis*, full-length AvrRps4 and variants were cloned into a Golden Gate–compatible pEDV3 vector with a C-terminal 4×myc tag.

### Protein Production and Purification.

Plasmids expressing the in planta processed C-terminal fragment of AvrRps4 (AvrRps4^C^) and integrated WRKY domain of RRS1 (RRS1^WRKY^) were expressed in *E. coli* SHuffle cells. The proteins were purified via IMAC followed by size-exclusion chromatography. Purified fractions were pooled and concentrated to 15 mg/mL and used for further studies. Detailed procedures are provided in *SI Appendix*, *Materials and Methods*.

### Crystallization and Structure Determination.

Crystals of the AvrRps4^C^–RRS1^WRKY^ complex were obtained from a 1:1 solution of 15 mg/mL protein with 0.8 M potassium sodium tartrate tetrahydrate, 0.1 M sodium Hepes (pH 7.5). Diffraction data were collected at the Diamond Light Source on the i03 beamline and processed in the P6_1/5_22 space group. The structure was determined by molecular replacement using the model of a monomer of AvrRps4^C^ (PDB ID code 4B6X) and the RRS1^WRKY^ from the PopP2–RRS1^WRKY^ complex (PDB ID code 5W3X) as the search model. Further details are provided in *SI Appendix*, *Materials and Methods*. X-ray data collection and refinement statistics are summarized in *SI Appendix*, Table S2.

### In Vitro Protein–Protein Interaction Studies.

AvrRps4^C^–RRS1^WRKY^ complex formation in vitro was studied using analytical gel filtration chromatography and ITC. The effect of structure-guided mutations on the AvrRps4^C^–RRS1^WRKY^ interaction in vitro was investigated using ITC as described in *SI Appendix*, *Materials and Methods*.

### Transient Cell-Death Assays and Coimmunoprecipitation Studies.

*Agrobacterium*-mediated transient cell-death assays were performed in *N. tabacum* and coimmunoprecipitation assays were performed in *N. benthamiana*. Detailed information concerning plant materials, growth conditions, plasmid construction, and immunoblotting are provided in *SI Appendix*, *Materials and Methods*.

### *Arabidopsis* HR Assays and Bacterial Growth Assays.

Bacterial strains *P. fluorescens* Pf0-EtHAn and *Pto* DC3000 were used for HR or in planta bacterial growth assays, respectively. The *Arabidopsis* accessions Ws-2 and Col-0 were used as wild type for all the assays in this study. Further details about plant materials, growth conditions, plasmid construction and mobilization, pathogen infection assays, and bacterial growth assays are provided in *SI Appendix*, *Materials and Methods*.

### WRKY–W-Box DNA Interaction: EMSA.

Complementary single-stranded DNA fragments encoding the W-box DNA sequence (forward strand: 5′-CGCCTTTGACCAGCGC-3′) were synthesized by IDT. EMSAs were performed using a Cy3-labeled W-box DNA probe in a reaction buffer containing 10 mM Tris⋅Cl (pH 7.5), 50 mM KCl, 1 mM dithiothreitol, and 5% glycerol as described in *SI Appendix*, *Materials and Methods*.

### WRKY–W-Box DNA Interaction: SPR Assay.

Complementary single-stranded DNA fragments encoding the W-box DNA sequence were synthesized by IDT. For SPR assays, the forward strand encoded the W-box DNA sequence (5′-CGCCTTTGACCAGCGC-3′) while the complementary reverse strand added an extra 20-bp ReDCaT sequence (5′-CCTACCCTACGTCCTCCTGC-3′) to complement the linker DNA added to the SA chip. The double-stranded DNA was then diluted to a working concentration of 1 μM. SPR measurements were performed at 25 °C using the reusable DNA capture technique (ReDCaT) as described (59, 60) and using a Biacore 8K System (Cytiva). Further details are provided in *SI Appendix*, *Materials and Methods*.

## Supplementary Material

Supplementary File

Supplementary File

Supplementary File

## Data Availability

All study data are included in the article and/or supporting information.
