# Supplementary material for: Perception of structurally distinct effectors by the integrated WRKY domain of a plant immune receptor
Source: Proc Natl Acad Sci U S A. 2021 Dec 8;118(50):e2113996118. doi: 10.1073/pnas.2113996118 (PMC8685902; doi:10.1073/pnas.2113996118)
Supplement: Supplementary File [file pnas.2113996118.sapp.pdf]

Supplementary information

Materials and Methods

Protein production and purification

Gene cloning, expression, and purification of proteins for in vitro binding studies

DNA encoding AvrRps4^{C} (134–221) was cloned in pOPIN-F (with a cleavable 6xHis-tag) expression vector while DNA fragments of RRS1^{WRKY} (1194-1273), RRS1B^{WRKY} (Asn1164-Thr1241), and *At*WRKY41 (Thr125-Ile204) were cloned in pOPIN-M (with a cleavable 6xHis-MBP-tag) expression vector using in-fusion cloning strategy (Clontech, Mountain View, CA, United States) (1). The constructs were then transformed in *Escherichia coli* (*E. coli*) SHuffle cells for expression. Bacterial cultures were grown in LB media (with 100 μg/mL carbenicillin) at 30°C to an OD_{600} = 0.6 followed by induction with 1 mM IPTG (isopropyl β-D-1-thiogalactopyranoside) and overnight growth 18°C. The cells were harvested by centrifugation at 6,000 g for 10 min and resuspended in Buffer A1 (50mM HEPES pH (8.0), 50mM glycine, 500mM NaCl, 30 mM imidazole and 5% v/v glycerol, EDTA free protease inhibitor tablets [1 tablet/50mL of A1 buffer]) followed by lysis by sonication with VC 750 VibraCell™ (Sonics) at 40 % amplitude, 1 sec on/3 sec off pulse for 20 min on ice. Cell debris was removed by centrifugation at 45,000 g for 60 mins. Purification of the proteins was performed using an ÄKTA Xpress purification system following two-step programme comprising initial capture by immobilised metal affinity chromatography (IMAC) [Step elution by Buffer B1 – Buffer A1 supplemented with 500 mM imidazole] followed by gel filtration with Superdex 75 26/600 gel filtration column pre-equilibrated in Buffer A4 (20 mM HEPES pH 7.5, 150 mM NaCl) supplemented with 1 mM TCEP. Fractions under the elution peak from the gel filtration columns were assessed by SDS-PAGE for the presence of the purified proteins and were then pooled and treated with 3C protease (10 μg/mg fusion protein) overnight at 4°C to cleave the 6xHis/6xHis-MBP-tag respectively. Cleaved 6xHis and 6xHis-MBP tags were then separated from their respective digested protein samples by passing the samples through a Ni^{2+}-NTA column and collecting the flow-through and wash samples. These samples were assessed by SDS-PAGE, pooled and concentrated via 3 kDa cut-off spin concentrators before subjecting to a second round of size-exclusion with a Superdex 75 16/60 gel

filtration column pre-equilibrated in Buffer A4. Eluted samples were then concentrated via 3 kDa cut-off spin concentrators to a final concentration of 10-15 mg/mL (as calculated by Nanodrop (Thermofisher Scientific™ , NanoDrop™ One Microvolume UV-Vis Spectrophotometer) at A_{205}) and were aliquoted and flash frozen at -80°C for subsequent analysis.

Expression and purification of proteins for crystallization

For crystallization of the AvrRps4^{C}/RRS1^{WRKY} complex, AvrRps4^{C} was cloned in pOPIN-A to express untagged protein and pOPIN-M construct of RRS1^{WRKY} (with cleavable 6xHis-MBP-tag) was used as mentioned above. Both the constructs were transformed individually into *E. coli* SHuffle cells and expressed using the above-mentioned conditions. The bacterial cells expressing AvrRps4^{C} and RRS1^{WRKY} were then mixed together, lysed and the proteins were co-purified via IMAC followed by size-exclusion chromatography as mentioned above. Eluted fractions were assessed by SDS-PAGE. The presence of untagged AvrRps4^{C} in the RRS1^{WRKY} eluted fractions confirmed complex formation in vitro. The eluted complex was subjected to 3C protease cleavage overnight at 4°C followed by an IMAC step to remove the cleaved 6xHis-MBP-tag. Fractions containing the untagged complex were then pooled, concentrated, and subjected to final gel filtration chromatography. The purified complex was concentrated to 15 mg/mL, aliquoted and used for crystallization studies.

Crystallization, data collection and structure solution

For crystallization of the AvrRps4^{C}/RRS1^{WRKY} complex, the sitting drop vapour diffusion method was used. Potential crystallization conditions were explored using commercially available crystallization screens. All crystallization trials were setup in 96-well plates, using an Oryx nano robot (Douglas Instruments) at a concentration of 7.5 mg/mL and 15 mg/mL at 20°C. Crystals of the AvrRps4^{C}/RRS1^{WRKY} complex appeared after few weeks in a condition comprising 0.8M Potassium sodium tartrate tertrahydrate, 0.1 M Sodium HEPES pH 7.5 from the Morpheus™ screen (Molecular Dimensions). The crystals were snap frozen in liquid nitrogen and shipped to Diamond Light Source for X-ray data collection.

Diffraction data was collected at Diamond Light Source, i03 beamline, under proposal mx18565. The data were scaled and merged by Aimless in the CCP4i2 software package (2). The AvrRps4^{C}/RRS1^{WRKY} complex structure was solved by molecular replacement using PHASER (3) with the structures of AvrRps4^{C} (PDB ID: 4B6X) and PopP2/RRS1^{WRKY} (PDB ID: 5W3X) as search models. Iterative cycles of manual model building using COOT (4) and ISOLDE (5) and refined using REFMAC (6) produced the final structure, which was then validated using MolProbity (7). Interaction interfaces were analyzed using PdbePISA (8). Models were visualized using ChimeraX (9) . The final protein model, and the data used to derive it, can be found in Protein Data Bank (PDB) (https://www.ebi.ac.uk/pdbe/) with the PDB ID: 7P8K.

Circular dichroism spectroscopy

Purified AvrRps4^{C} wild-type and mutants were dialyzed in 10mM phosphate buffer, pH 8.0 at a final concentration of 0.5 mg/mL. Samples were analyzed in the far-UV region between 190-260 nm at 20°C by using Chirascan™ plus CD Spectrometer (Applied Photophysics) and quartz cuvette of path length 1mm. For each sample, three successive spectral scans were averaged and adjusted by subtracting corresponding blanks. The results were plotted using ggplot2 in R (10)

In vitro Protein–Protein Interaction studies

Analytical gel filtration

To study AvrRps4^{C} and RRS1^{WRKY} complex formation in vitro, individual proteins (at a concentration of 1 mg/mL) were applied to pre-equilibrated (Equilibration buffer - 20 mM HEPES pH 7.5, 150 mM NaCl, 1 mM TCEP) Superdex 75 10/300 analytical column (GE-Healthcare) using an AKTA Explorer (GE-Healthcare) at 4°C and eluted at a flow rate of 0.5 mL/min by monitoring the absorbance at 280 nm. 500 μL fractions were collected and analyzed by SDS-PAGE. For complex formation, proteins were combined in a 1:1 molar ratio and incubated on ice for 1-2 hrs before the analysis. The results were plotted using ggplot2 in R (10)

Isothermal titration calorimetry (ITC)

ITC experiments were performed using a MicroCal PEAQ-ITC (Malvern, UK). To test the interaction of AvrRPS4^{C} wild-type or structure-guided mutants with RRS1^{WRKY}, *At*WRKY41 or RRS1B^{WRKY}, the calorimetric cell was filled with 20 μM of RRS1^{WRKY}/*At*WRKY41/RRS1B^{WRKY} and titrated with 200 μM of AvrRps4^{C} wild-type/mutants in the syringe. Each ITC run included a single injection of 0.5 μL followed by 18 injections of 2 μL each. Injections were made at 120-second intervals with a stirring speed of 750 rpm. Data were processed with AFFINImeter ITC analysis software (11). ITC runs for wild-type and mutants were done in triplicate at 25°C using buffer A4. All the ITC curves were plotted using ggplot2 in R (10).

Transient cell death assays and co-Immunoprecipitation studies

N. tabacum cell death assays

Transient cell death assays were performed using 4-5 week-old *N. tabacum* "Petit Gerard" as described previously (12). Plants were grown in long days (16 hr light/8 hr dark) under high light intensity at 24°C. *Agrobacterium tumefaciens* GV3101 was used to deliver C-terminal 4xmyc-tagged full-length constructs of AvrRps4 wild-type and mutants, and C-terminal 6xHis/3xFLAG-tagged RRS1-R, RRS1-S, RRS1B, RPS4, RPS4B. Agrobacterium cells expressing these constructs were grown at 28°C, harvested, and resuspended in infiltration buffer (10 mM $MgCl_2$, 10 mM MES [pH 5.6]), supplemented with 150 μM acetosyringone. Appropriate combinations of the above constructs were mixed at an $OD_{600} = 0.5$ per construct and were hand infiltrated on the abaxial surface of 4–5-week-old *N. tabacum* leaves by a 1ml needleless syringe. Infiltrated leaves were detached 5 days post infiltration (dpi) and imaged under white light. The experiment was done in triplicate with similar results.

In planta co-immunoprecipitation assays

For co-immunoprecipitation assays**,** proteins were transiently expressed in 4-5 week old *N. benthamiana* leaves using agroinfiltration as described in (13). Leaf samples (4 g) were harvested at 3 dpi, frozen in liquid nitrogen, and ground to fine powder. A total of 8 mL (two times weight/volume) of ice-cold protein extraction buffer [10% glycerol, 1 mM EDTA, 25 mM Tris

[pH 7.5], 150 mM NaCl, 2% w/v PVPP, 10 mM DTT, 1x protease inhibitor cocktail [Sigma], 1 % vol/vol Nonidet P-40] was added to the ground tissue and samples were centrifuged at 6,000 × g at 4°C for 15 min. Supernatant was then filtered through miracloth to remove residual plant debris. 50μL of the filtrate was aliquoted and ran on 4-20 % precast SDS-PAGE gels to check for the expression of the proteins in the input fraction. Residual samples were mixed with 50 μL Flag beads and incubated at 4°C (with constant rotation) for 1 hr. Flag beads were washed three times with IP buffer (10% glycerol, 1 mM EDTA, 25 mM Tris [pH 7.5], 150 mM NaCl, 1 % vol/vol Nonidet P-40) and resuspended in 30 μL SDS-loading buffer. Immunoprecipitated samples were recovered from the flag beads by boiling at 70°C for 10 min. Eluted samples were separated by 4-20% precast SDS-PAGE, electroblotted onto PVDF membranes (Bio-Rad), and probed with HRP-conjugated anti-FLAG M2 (1:10000 dilution, Sigma) and anti-Myc (1:5000, Santa Cruz) as required.

Arabidopsis HR assays and bacterial growth assays

Plant material and growth conditions

Arabidopsis accessions Ws-2 and Col-0 were used as wild-type for all the assays in this study. Ws-2 was the background of the triple mutant *rrs1-1/rps4-21/rps4b-1* while double mutant *rrs1-3/rrs1b-1* and single mutants *rrs1-3* and *rrs1b-1* lines were in the Col-0 background. The plants were grown under short day conditions (10-h light/14-h dark) at 22°C and 65% humidity for 4-5 weeks before being used for assays.

Arabidopsis HR assays

For Arabidopsis HR assays, Pf0-EtHAn was grown on King's B agar medium containing chloramphenicol (30 μg/ mL) at 28°C. Plasmids were mobilized into Pf0-EtHAn using tri-parental mating method using *E. coli* HB101 (pRK2013) as a helper strain as described in (14). For HR assays bacteria were grown overnight at 28°C in KB media and cells were harvested, washed and resuspended in freshly prepared sterile 10 mM $MgCl_2$. Final concentration of the inoculum was adjusted to OD_{600} = 0.3. The inoculum was then hand infiltrated into the leaves of 5-week-old plants with 1-ml needleless syringes. 5-6 leaves were infiltrated for each construct per genotype

per biological replicate. Plants were then blotted with the tissue to remove the excess bacteria and then kept at 22°C covered with a transparent dome. HR was scored 20 hrs post infection. The experiment was repeated three times with similar results.

Bacterial growth assay

Pto DC3000 containing full-length 4xc-myc-tagged wild-type AvrRps4, or structure guided AvrRps4 mutant variants and the AvrRps4 KRVY/AAAA mutant (negative control) were grown on selective King's B (KB) medium plates (containing 50 μg/mL Rifampicin and 20 μg/mL Gentamycin) for 48 h at 28°C. Bacterial cells were harvested, washed and resuspended in sterile 10 mM $MgCl_2$ to a final OD_{600} = 0.001. The bacterial suspension was then hand infiltrated on the abaxial surface of 5-week-old Arabidopsis leaves using a 1mL needleless syringe. For the bacterial growth assays, 2 leaves each of 10 independent plants/genotype/construct constitute one biological replicate with three replicates in total. Samples from 4 plants were collected at day 0 and samples from 6 plants were collected at day 3. For quantification, 2 leaf discs from one plant (one leaf disc per leaf) were collected with a 6-mm-diameter cork borer (disc area - 0.283 cm^2) and were ground in 200 μL of infiltration buffer (10 mM $MgCl_2$). For day 0, samples from 4 plants were independently ground and spotted (10 μL /spot) on selective KB medium. For day 3, samples from 6 plants were independently ground, serially diluted (5, 50, $5X10^2$, $5X10^3$ and $5X10^4$ times) and spotted (6 μL/spot) on selective KB medium. The plates were incubated at 28°C for two days before colony forming units (CFU/drop) were calculated. Bacterial growth is represented as CFU cm^{-2} of leaf tissue. Statistical significance was determined by one-way ANOVA followed by post-hoc Tukey HSD analysis. The results were plotted using ggplot2 in R (10).

WRKY/W-box DNA interaction

Electrophoretic mobility shift assay (EMSA assay)

Complimentary single-stranded DNA fragments containing W-box DNA sequence (forward strand: CGCCTTTGACCAGCGC) were synthesized by IDT. The forward strand was labelled with Cy3 probe at the 5'end. These strands were annealed by mixing 55 μL of the forward strand and 45 μL of the reverse strand and heating to 90 °C for 10 min and then cooling to 4°C. For the titrations, protein samples were prepared by mixing 1 μM of $RRS1^{WRKY}$ or *At*WRKY41 with

varying amounts of AvrRps4^C or AvrRps4^C EE/AA mutant (0, 0.1, 0.2, 0.5, 1, 2, 3, 5 μM) in a reaction volume of 20 μL containing reaction buffer 10 mM Tris-Cl pH 7.5, 50 mM KCl, 1mM DTT, 5 % Glycerol. The samples were incubated on ice for 30 min. 1 nM of Cy3 labelled dsDNA containing W-box fragment or scrambled DNA was then added into the reaction mixture. Samples were incubated on ice for 30 min before gel electrophoresis. The samples were loaded onto 10% non-denaturing polyacrylamide gels and run at 4°C in 0.5 × TBE buffer. The gels were imaged under UV light.

Surface Plasmon Resonance (SPR) based ReDCaT chip assay

Complimentary single-stranded DNA fragments containing W-box DNA sequence were synthesized by IDT. The forward strand contained the W-box DNA sequence (CGCCTTTGACCAGCGC) while the complimentary reverse strand contained an additional 20 bp ReDCaT linker sequence (5′-CCTACCCTACGTCCTCCTGC-3′) complimentary to the linker sequence on the SA-chip. The single stranded DNA fragments were dissolved to a final concentration of 100 μM in water. These strands were annealed by mixing 55 μL of the forward strand and 45 μL of the reverse strand (containing the ReDCaT linker) and heating to 90°C for 10 mins then cooling to 4°C. The dsDNA was diluted to a working concentration of 1 μM in SPR running buffer (10 mM HEPES pH 7.5, 250 mM NaCl, 0.005% Tween-20). SPR measurements were performed at 25°C using the Reusable DNA Capture Technique (ReDCaT) as described in (15) and using a Biacore 8K system (Cytiva). Briefly, ReDCaT uses a sensor chip SA (Cytiva) to which a 20 bp biotinylated ReDCaT linker is immobilized to the test flow cell only. The W-box DNA fragment containing the complimentary ReDCaT linker sequence was then immobilized onto the chip by flowing over the ReDCaT chip (both test and reference flow cells) at a flow rate of 10 μL/min. To test the binding of RRS1^{WRKY} or *At*WRKY41 was flowed over both reference and test flow cells on the ReDCaT chip, followed by SPR running buffer to observe any dissociation from the W-box DNA.

To test the effect of AvrRps4^C or AvrRps4^C EE/AA on the RRS1^{WRKY} or *At*WRKY41 DNA binding, 2 μM of RRS1^{WRKY} or *At*WRKY41 was incubated with the increasing concentration of AvrRps4^C or AvrRps4^C EE/AA (0.1, 0.2, 0.5, 1, 2, 3, 5 μM) at 4°C for 30 min. The samples were

then injected over the W-box DNA (and reference flow cell) and the signal recorded. The chip was regenerated between each run using 1 M NaCl and 50 mM NaOH to remove the DNA with any residual bound protein. The experiment was performed in eight replicates across the 8 channels and the responses referenced against a reference flow cell. The recorded sensorgrams were analyzed using Biacore Insight Evaluation software version 3.0.11.15423 (Cytiva).

The SPR signal was recorded as response units (RUs) and data was normalized by correcting for size of DNA, DNA capture, and protein molecular weight. % Rmax is calculated is calculated as:

$$Rmax = RU_{obs}/Rmax_{theoretical} \text{ X } 100$$

Where RU_{obs} is the measured response just prior to the end of the injection and $R_{max\ theoretical}$ is the expected theoretical response for the interaction which can be calculated using following equation :

$$R_{max} = M\ wt_{protein}/\ Mwt_{DNA} \text{ X } R_L \text{ X } n \text{ X } 0.78$$

Where Mwt is the molar mass, R_L is the DNA capture response, 0.78 is a constant used for estimating responses for DNA/protein interactions and n is the binding stoichiometry. Data was plotted using ggplot in R (10).

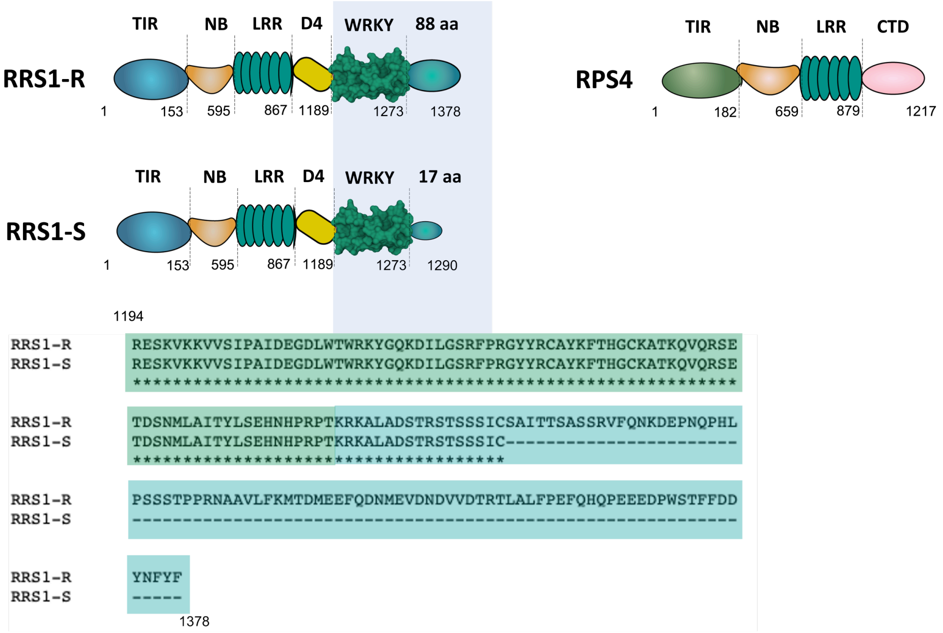

Fig. S1. Schematic representation of the domain organization of RRS1-R/RRS1-S and RPS4. Predicted domain boundaries of RRS1-R/RRS1-S/RPS4 are shown. The sequence alignment of the RRS1^{WRKY} + C-terminal domain in RRS1-R and RRS1-S reveals a truncated C-terminal domain in RRS1-S after the WRKY domain. The WRKY domain is highlighted in green, whereas the C-terminal domain is highlighted in blue.

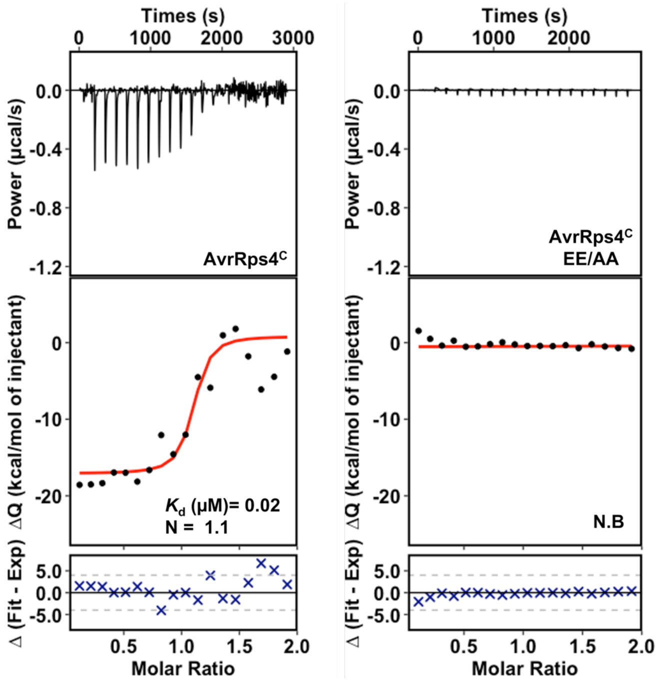

Fig. S2. AvrRps4^{C} interacts with *At*WRKY41 in vitro. Isothermal titration calorimetry (ITC) of *At*WRKY41 with wild-type AvrRps4^{C} and AvrRps4^{C} E175A/E187A (EE/AA) mutant. Raw processed thermogram after baseline correction and noise removal is displayed in the upper panel. The lower panel represents the experimental binding isotherm obtained for the interaction of AvrRps4^{C} and mutant with *At*WRKY41 together with the global fitted curves (displayed in red) obtained from three independent experiments using AFFINImeter software (11). The K_d was derived from fitting to a 1:1 binding model.

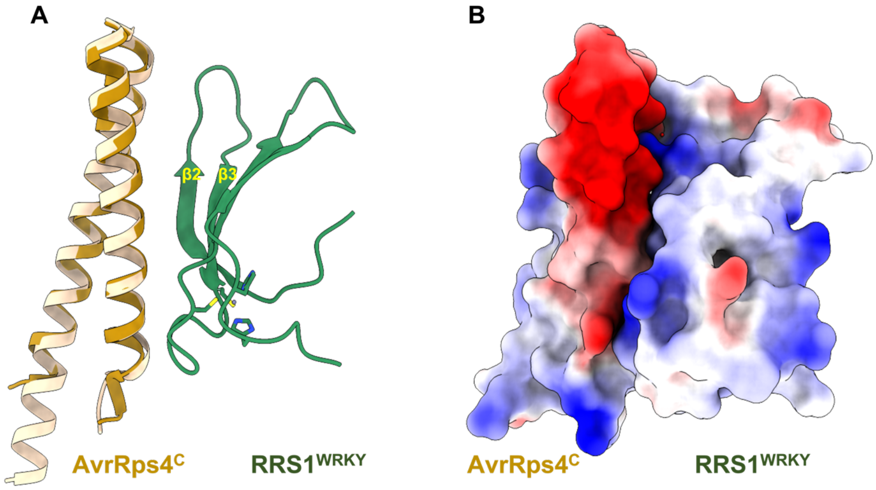

Fig. S3. (A) Overlay of the crystal structure of AvrRps4^{C}/RRS1^{WRKY} (Dark goldenrod/Dark green) with the previously published crystal structure of AvrRps4^{C} (light goldenrod) (PDB ID: 4B6X). (B) Electrostatic surface representation of the AvrRps4^{C}/RRS1^{WRKY} complex highlighting the electronegative patch in AvrRps4^{C} and electropositive patch in RRS1^{WRKY} at the interface.

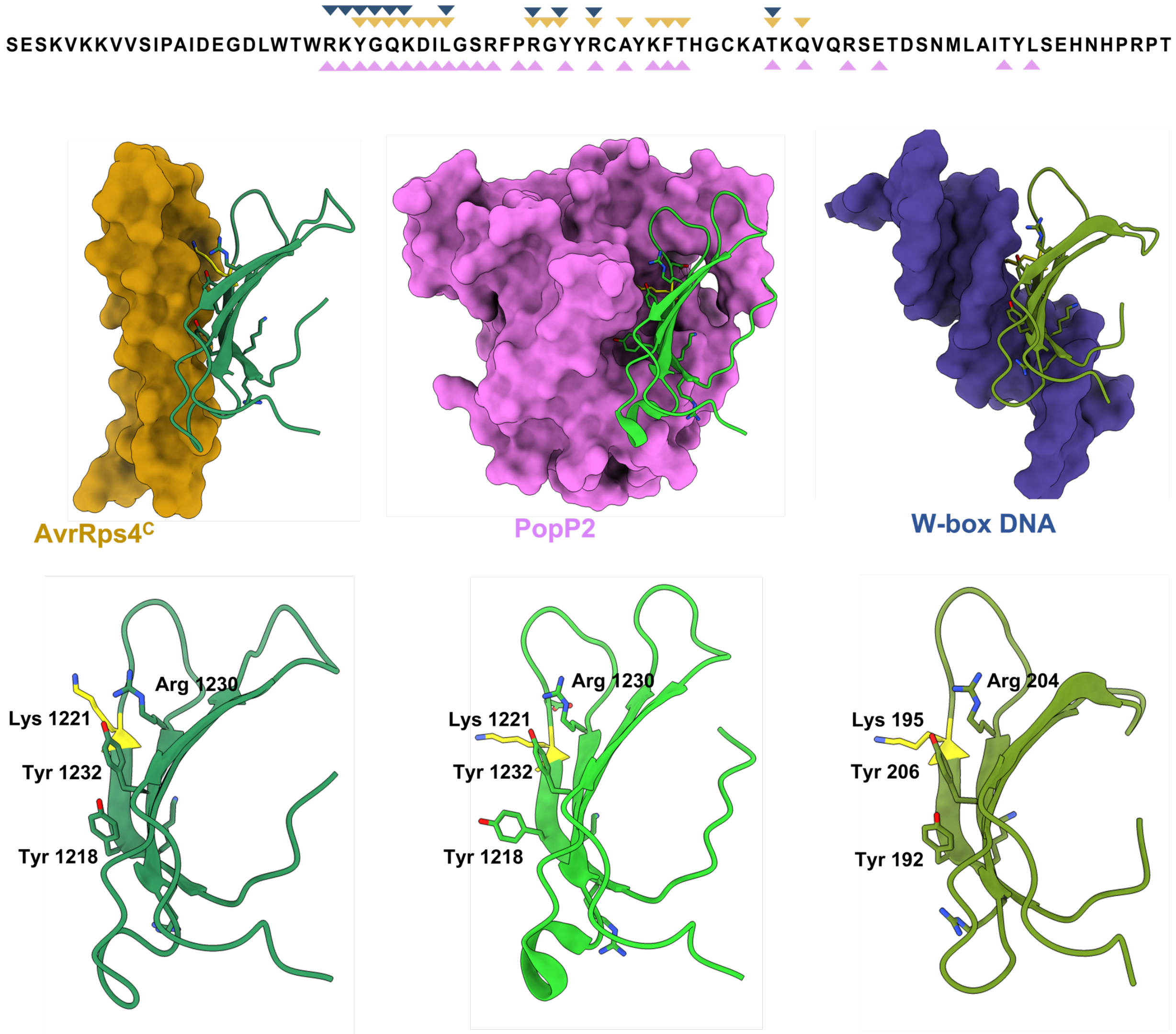

Fig. S4. Comparative views of the AvrRps4^{C}, PopP2, and W-box DNA binding region of WRKY domains. The upper panel shows RRS1^{WRKY} residues buried in the RRS1^{WRKY}/AvrRps4^{C} complex (gold triangles), RRS1^{WRKY}/PopP2 complex (purple triangles) and corresponding residues in *At*WRKY33/W-box DNA complex (blue triangles). The middle panel shows cartoon representations of the crystal structure of AvrRps4^{C}/RRS1^{WRKY} (gold (surface)/sea green (ribbon)), PopP2/RRS1^{WRKY} (PDB: 5W3X, purple (surface)/lime green (ribbon)) and W-box DNA/*At*WRKY33-N complex (PDB: 6J4G, blue(surface)/forest green (ribbon)). The lower panel depicts cartoon representations of the respective WRKY domains with residues mediating the interaction with its cognate partner highlighted. Lys1221 (in RRS1^{WRKY}) and the corresponding residue in *At*WRKY33-N are highlighted in yellow.

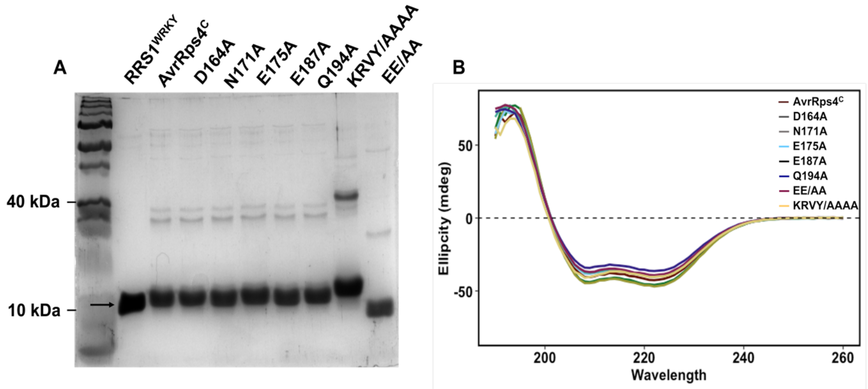

Fig. S5. (A) SDS-PAGE of RRS1^{WRKY} and AvrRps4^{C} (wild-type and mutants) samples used for in vitro studies. Arrow indicates the expected size of the purified proteins. (B) CD spectra of the wild-type AvrRps4^{C} and mutants. Far-UV spectra corresponding to the wild-type (brick red), D164A (dark green), N171A (olive green), E175A (cyan), E187A (bluish green), Q194A (dark blue), EE/AA (purple) and KRVY/AAAA (coral) are shown. Spectra were taken at 20°C using 0.5 mg/mL of each protein. Each scan represents the average of three independent measurements.

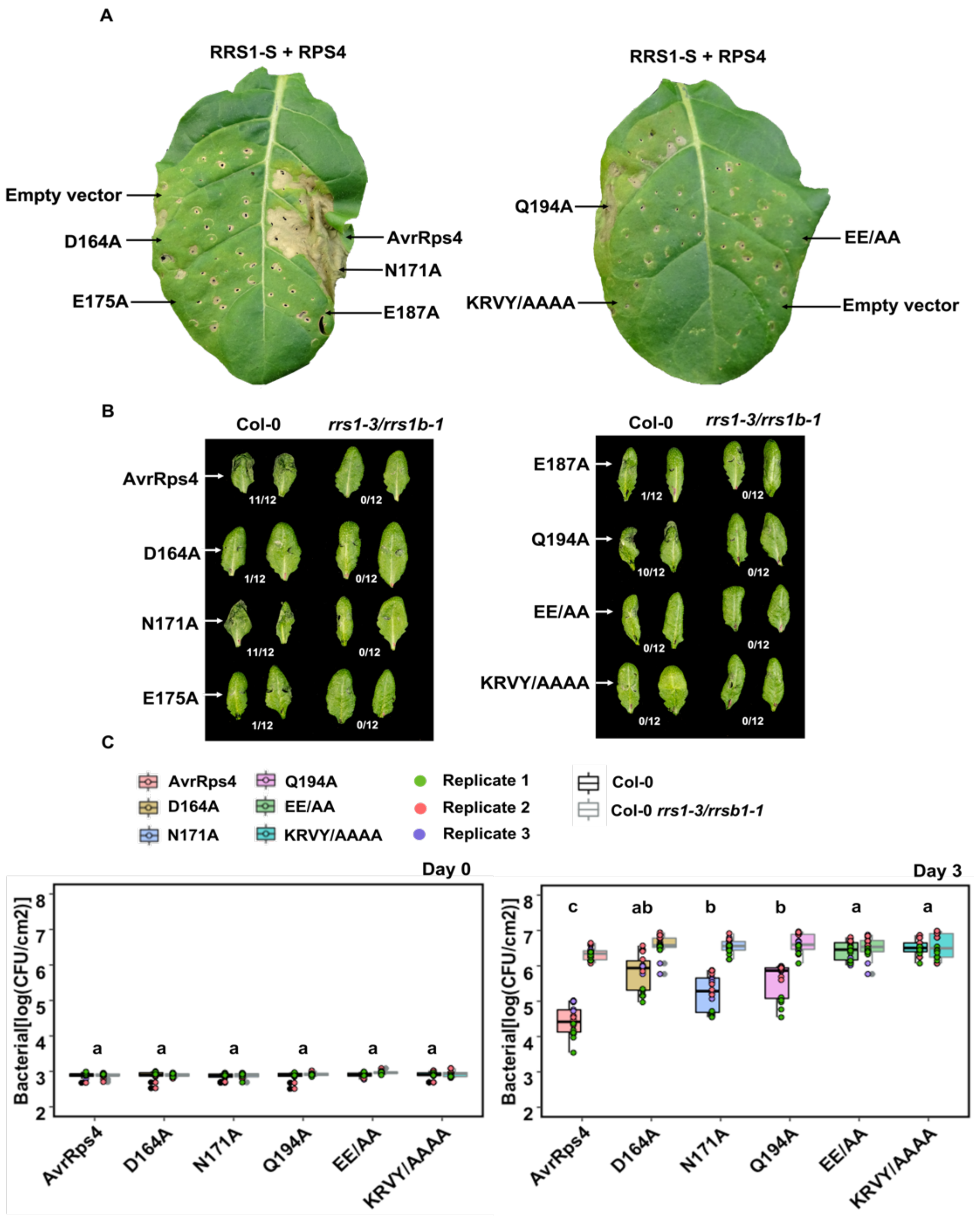

Fig. S6. Structural guided mutants of AvrRps4 confer distinct RRS1-S/RPS4 dependent recognition specifies. (A) Structure-guided mutants in AvrRps4 at the AvrRps4^{C}/RRS1^{WRKY} interface compromise RRS1-S/RPS4 mediated cell death in *N. tabacum*. Representative leaf images show RRS1-S/RPS4 mediated cell death response to wild-type and a subset of structure-guided mutants of AvrRps4. Agroinfiltration assays were performed in 4-5-week-old *N. tabacum* leaves, and cell death was assessed at 4 dpi. The experiment was repeated three times with similar results. (B) Hypersensitive response (HR) assay in Arabidopsis lines using *Pseudomonas*

fluorescens (*Pf*) Pf0-1 secreting AvrRps4 wild-type and mutants. Constructs were delivered from (*Pf*) Pf0-1 into Arabidopsis Col-0 and Col-0 *rrs1-3/rrsb-1* background and HR observed 20 hours post-infiltration. Fraction refers to number of leaves showing HR of 12 randomly inoculated leaves. This experiment was repeated at least three times with similar results. (C) In planta bacterial growth assays with Pto DC3000 secreting AvrRps4 wild-type and mutants on Col-0 and Col-0 *rrs1-3/rrsb-1* background plants. Bacterial suspensions with OD_{600} = 0.001 were pressure infiltrated into the leaves of 5-week-old Arabidopsis plants. Values are plotted from three independent experiments (shown in different colors). Statistical significance of the values was calculated by one-way ANOVA followed by post-hoc Tukey HSD analysis. Letters above the data points denotes significant differences ($P<0.05$). Detailed statistical summary can be found in Table 5.

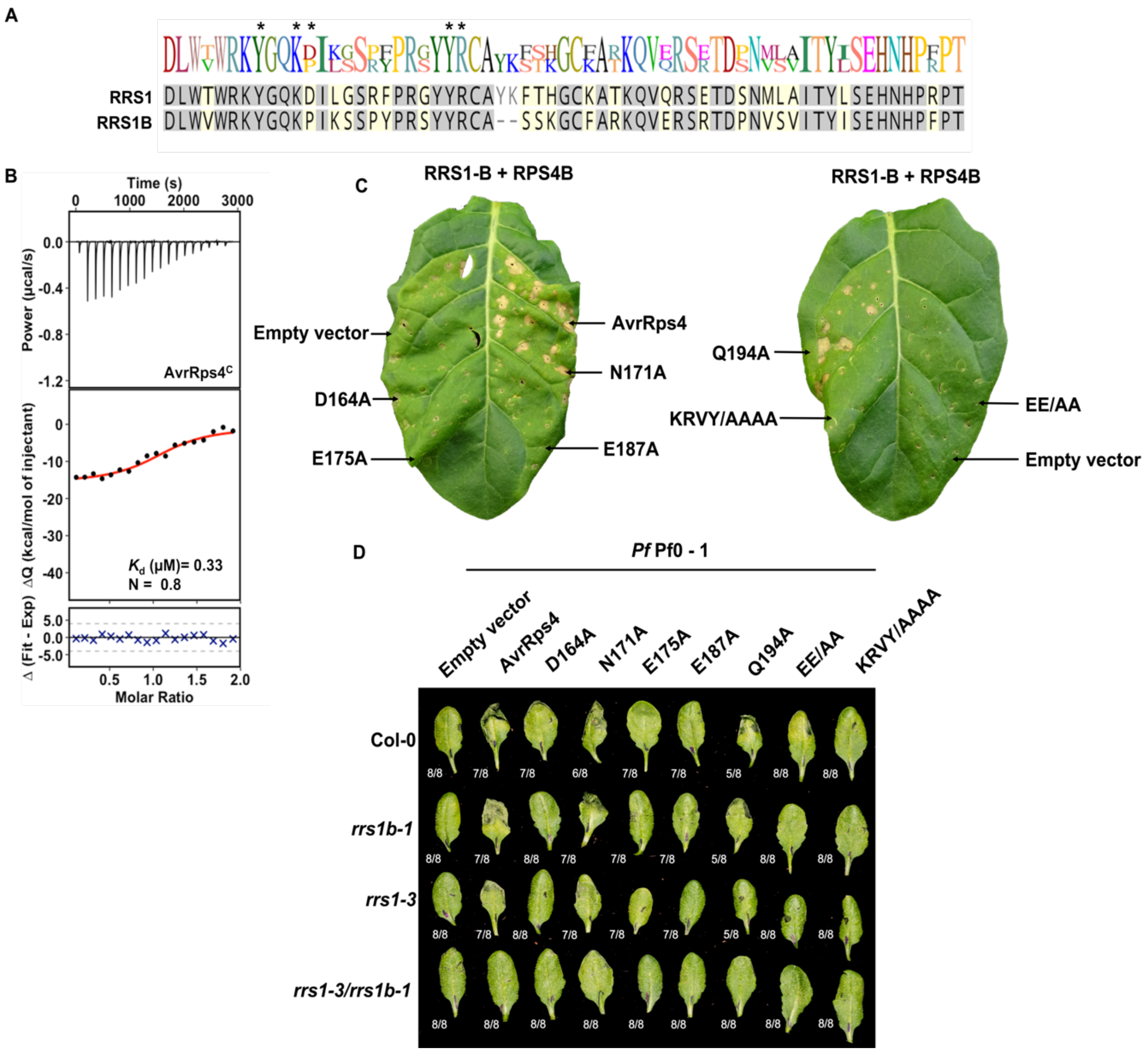

Fig. S7. (A) Protein sequence alignment of the integrated WRKY domains from RRS1 and RRS1B. Grey shaded letters show identical residues. Residues at the AvrRps4^{C}/RRS1^{WRKY} interface are marked with asterisk. (B) Isothermal titration calorimetry (ITC) titrations of RRS1B^{WRKY} with wild-type AvrRps4^{C}. Raw processed thermogram after baseline correction and noise removal is displayed in the upper panel. The lower panel shows the experimental binding isotherm for the interaction together with the global fitted curve (displayed in red) obtained from three independent experiments using AFFINImeter software (11). The K_d was derived from fitting to a 1:1 binding model. (C) Structural guided mutants of AvrRps4 at the AvrRps4^{C}/RRS1^{WRKY} interface compromise RRS1B/RPS4B mediated cell death responses in *N. tabacum*. Representative leaf images show RRS1B/RPS4B mediated cell death response to AvrRps4 wild-

type and a subset of structure-guided mutants. Agroinfiltration assays were performed in 4- to 5-week-old *N. tabacum* leaves, and HR phenotypes were assessed at 4 dpi. The experiment was repeated three times with similar results. (D) Hypersensitive response (HR) assay in different Arabidopsis lines using *Pseudomonas fluorescens* (*Pf*) Pf0-1 secreting AvrRps4 wild-type and mutants. Constructs were delivered from Pf0-1 into Arabidopsis Col-0, Col-0 *rrs1-3*, Col-0 *rrs1b-1*, Col-0 *rrs1-3*/*rrs1b-1* background and HR was observed 20 hours post-infiltration. Fraction refers to number of leaves showing HR of 8 randomly inoculated leaves. This experiment was repeated twice with similar results.

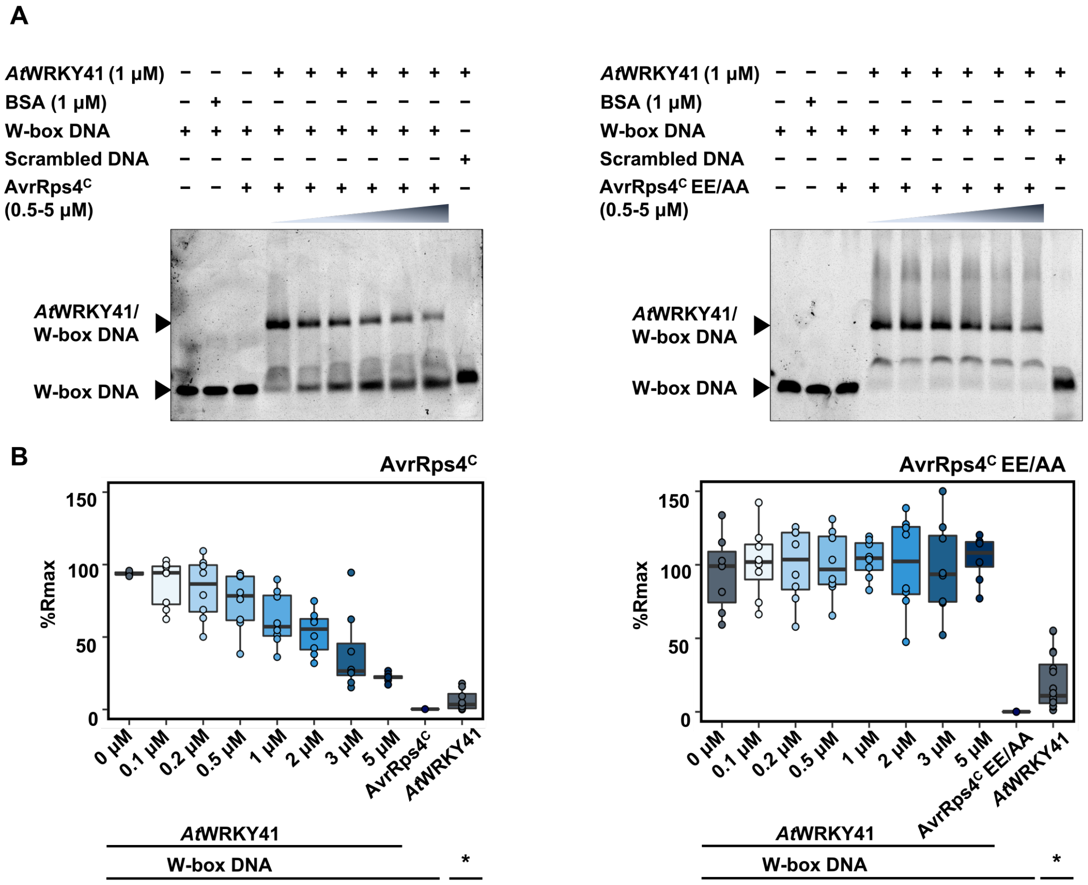

Fig. S8. AvrRps4 interferes with W-box DNA binding by *At*WRKY41. (A) Electrophoretic mobility shift assay (EMSA) of the DNA binding activity by *At*WRKY41 in the presence of increasing concentrations of AvrRps4^{C} or AvrRps4^{C} EE/AA mutant. BSA was used as a negative control for W-box DNA binding. Scrambled DNA was used as a negative control to test the specificity of *At*WRKY41 to W-box DNA. The experiment was repeated three times with similar results. B) SPR based ReDCaT chip assay was performed using W-box and scrambled DNA (used as a negative control). Percentage of normalized response (% Rmax) of *At*WRKY41 binding to W-box DNA and scrambled DNA immobilised on a ReDCaT SPR chip. Titrations were performed following pre-incubation of *At*WRKY41 with increasing concentrations of AvrRps4^{C} or AvrRps4^{C} EE/AA mutant. The experiment was performed in eight replicates (each dot represents one replicate).

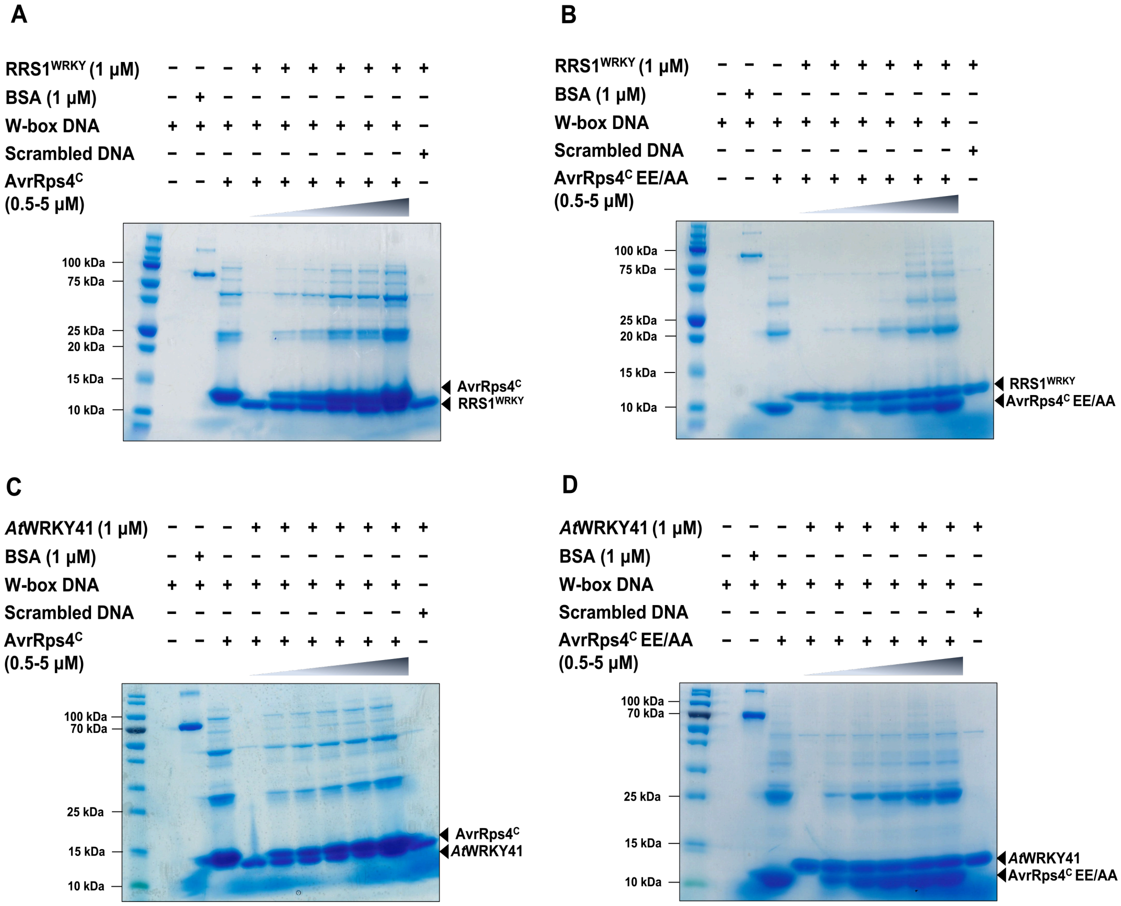

Fig. S9. SDS-PAGE of the samples used in the EMSA assays. Top: Titration experiment between RRS1^{WRKY} and W-box DNA with increasing concentrations of (A) AvrRps4^{C} and (B) AvrRps4^{C} EE/AA mutant. Bottom: Titration experiment between *At*WRKY41 and W-box DNA with increasing concentration of (C) AvrRps4^{C} and (D) AvrRps4^{C} EE/AA mutant.

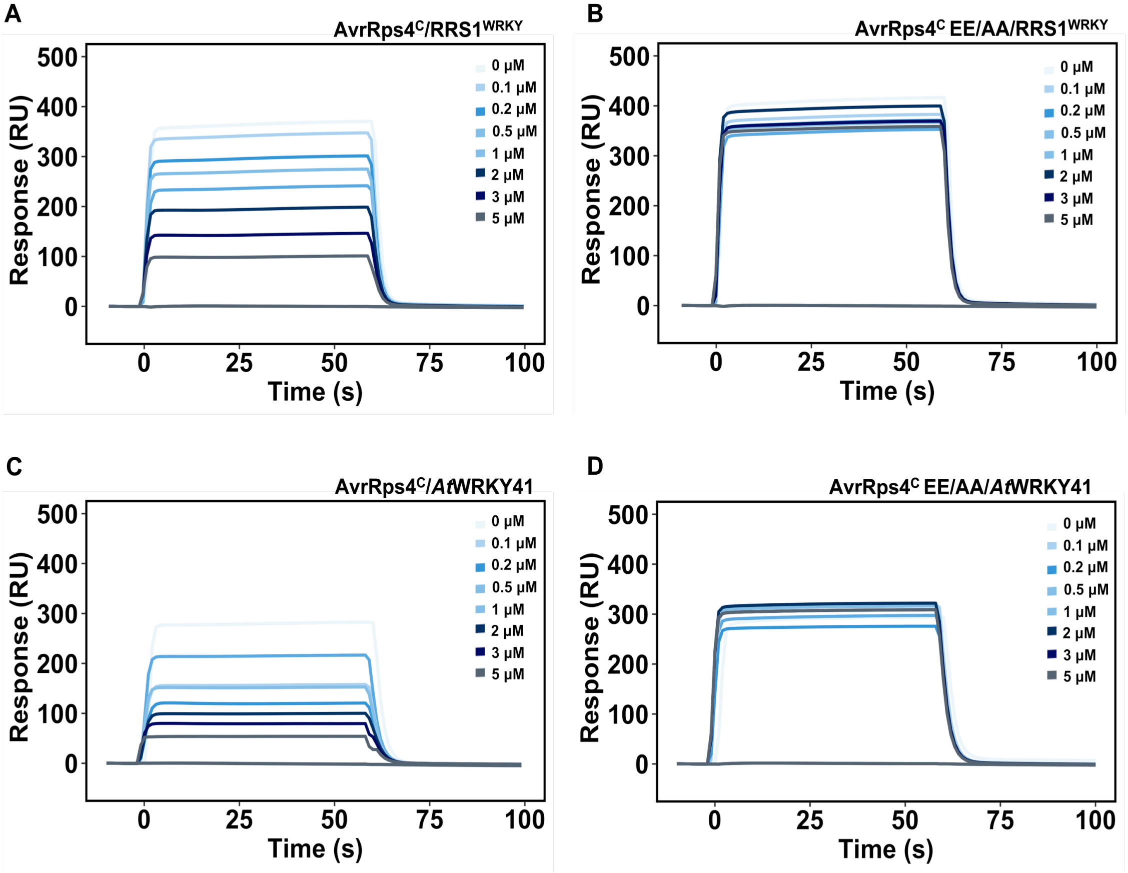

Fig. S10 Raw sensogram obtained from the binding of RRS1^{WRKY}/*At*WRKY41 on W-box DNA immobilised on a ReDCaT SPR chip with increasing concentration of AvrRps4^{C}/AvrRps4^{C} EE/AA.

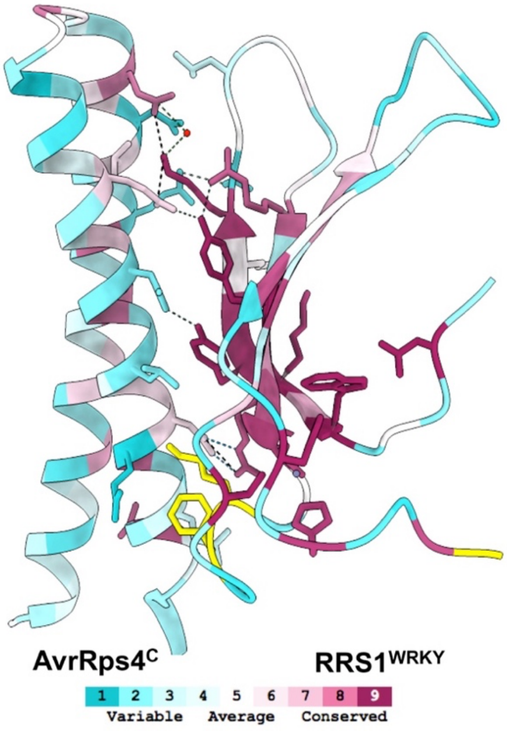

Fig. S11. ConSurf analysis for the interface of the AvrRps4^{C}/RRS1^{WRKY} complex. The conservation profiles of residues mediating the interaction of AvrRps4^{C} and RRS1^{WRKY} as calculated by Consurf are mapped upon the structures (16). The complex is shown in cartoon representation and residues mediating interaction at the interface are highlighted. Each segment of the cartoon has been colored according to its conservation status ranging from purple (highly conserved) through white (moderately conserved) to cyan (highly variable). Segments highlighted in yellow are residues for which a meaningful conservation level could not be derived from the set of homologues sequences used for the analysis.

Movie S1. Crystal structure of the AvrRps4^{C}/RRS1^{WRKY} complex. **(0-1sec)** Cartoon representation of AvrRps4^{C} and RRS1^{WRKY} complex. AvrRps4^{C} is shown in gold and RRS1^{WRKY} is shown in green (including the zinc atom and zinc-binding residues). **(2-10 sec)** Electrostatic surface representation of AvrRps4^{C} in the AvrRps4^{C}/RRS1^{WRKY} complex displaying a prominent negative patch on AvrRps4^{C} and electropositive patch on RRS1^{WRKY} at the interface. **(11-23 sec)** Close-up view of the interface displaying residues in RRS1^{WRKY} that interact with the electronegative patch of AvrRps4^{C}. The side chain of RRS1^{WRKY} Lys1221 (that is acetylated by PopP2) is shown in yellow and protrudes into the acidic cleft on the surface of AvrRps4^{C}. **(12-33 sec)** Overall structure of AvrRps4^{C}/RRS1^{WRKY} highlighting all the residues at the interface.

Movie S2. Comparison of the AvrRps4^{C}, PopP2, and W-box DNA binding region of WRKY domains. (0-8 sec) Cartoon representation of the crystal structure of AvrRps4^{C}/RRS1^{WRKY} (gold (surface)/green (ribbon), including the zinc atom and zinc-binding residues). Residues of RRS1^{WRKY} that bind AvrRps4^{C} are also shown. The side chain of RRS1^{WRKY} Lys1221 is shown in yellow which protrudes into a pocket on the surface of AvrRps4^{C}. **(9-22 Sec)** Overlay of the crystal structure of AvrRps4^{C}/RRS1^{WRKY} with PopP2/RRS1^{WRKY} (PDB: 5W3X, purple (surface)/lime green (ribbon)) illustrating how RRS1^{WRKY} perceives AvrRps4^{C} and PopP2 using the same interface. **(23-38 sec)** Overlay of the crystal structure of AvrRps4^{C}/RRS1^{WRKY} with *At*WRKY33/W-box DNA (PDB: 6J4G, blue (surface)/forest green (ribbon)) displaying the overlapping interface used by the WRKY domain to bind AvrRps4^{C} and W-box DNA.

Table 1. Thermodynamic parameters obtained from ITC experiments

**Cell**		**Syringe**		**T**	**∆H**	K_d
**Contents**	**Conc. [mM]**	**Contents**	**Conc. [mM]**	**[^{0}C]**	[kcal mol^{-1}]	[M^{-6}]
RRS1^{WRKY}	0.02	AvrRps4^{C}	0.2	25	-40 ± 0.036	0.10
RRS1^{WRKY}	0.02	D164A	0.2	25	n.b.	n.b.
RRS1^{WRKY}	0.02	N171A	0.2	25	-25.42 ± 0.02	0.04
RRS1^{WRKY}	0.02	E175A	0.2	25	n.b.	n.b.
RRS1^{WRKY}	0.02	E187A	0.2	25	n.b.	n.b.
RRS1^{WRKY}	0.02	Q194A	0.2	25	-19.89 ± 0.25	0.70
RRS1^{WRKY}	0.02	EE/AA	0.2	25	n.b.	n.b.
RRS1^{WRKY}	0.02	KRVY/AAAA	0.2	25	-19.59 ± 0.02	0.03
RRS1B^{WRKY}	0.02	AvrRps4^{C}	0.2	25	-14.48 ± 0.01	0.33
*At*WRKY41	0.02	AvrRps4^{C}	0.2	25	-90.54 ± 0.44	0.02
*At*WRKY41	0.02	AvrRps4EE/AA	0.2	25	n.b.	n.b.

Table 2. Data collection and refinement statistics for the crystal structure of the AvrRps4^{C}/RRS1^{WRKY} complex.

*The highest resolution shell is shown in parenthesis.
**As calculated by MolProbity

**Data collection statistics**	
Wavelength (Å)	0.912
Space group	$P\ 6_1\ 2\ 2$
Cell dimensions a, b, c (Å)	105.61, 105.61, 66.99
Resolution (Å)*	45.77 - 2.65 (2.78 - 2.65)
R_{merge} (%)	9.5 (119.0)
(I)/σ(I)	17.4 (2.3)
Completeness (%)	99.9 (100)
Unique reflections	6800 (888)
Redundancy	20.8 (21.9)
$CC^{1/2}$	1.0 (0.9)
**Refinement and model statistics**	
Resolution (Å)*	45.77 - 2.65 (2.72 - 2.65)
R_{work} / R_{free} (%)	24.5 (35.3) / 28.3 (45.1)
No. atoms	1020
Protein	1016
Water	3
Mean B value (overall Å^2)	93.11
rmsd bond lengths (Å)	0.0039
rmsd bond angles (°)	1.24
Ramachandran plot (%)**	
Favoured	95.9%
Allowed	4.1%
Outliers	0
MolProbity Score	1.89 (98th percentile)

Table 3. Interface summary for AvrRps4^C/RRS1^{WRKY} complex. Interface analysis was performed using PDBePISA

	AvrRps4^C		RRS1^{WRKY}	
**Number of residues**				
Interface	15	24.20%	15	23.40%
Surface	62	100.00%	64	100.00%
Total	62	100.00%	64	100.00%
Solvent-accessible area, Å				
Interface	589.7	12.30%	593.9	11.90%
Total	4787.5	100.00%	5006.9	100.00%
**Solvation energy, kcal/mol**				
Isolated structure	-36.5	100.00%	-49.9	100.00%
Gain on complex formation	-1.6	4.50%	-2.4	4.80%
Average gain	-0.7	1.90%	-1	2.00%
P-value	0.401		0.327	

**Interface area (Å^2)**	591.8
**Solvation energy (kcal/mol)**	-4
**Hydrophobic P-value**	0.362
**Hydrogen bonds**	10
**Salt bridges**	7
**Disulphide bonds**	0
**CSS**	0.1

Table 4. **Interaction summary of the residues mediating the intermolecular contacts between AvrRps4^{C} and RRS1^{WRKY}**

**Hydrogen bonds**			
**AvrRps4^{C}**		**Dist. [Å]**	**RRS1^{WRKY}**
1	A:ASN 171[ND2]	2.68	B:TYR1218[OH]
2	A:ASN 190[ND2]	2.93	B:ASP1222[O]
3	A:GLN 194[NE2]	2.99	B:ASP1222[O]
4	A:GLU 187[OE2]	3.32	B:LYS1221[NZ]
5	A:THR 191[OG1]	3.00	B:LYS1221[NZ]
6	A:GLN 194[OE1]	2.90	B:ASP1222[N]
7	A:GLU 175[OE2]	3.24	B:ARG1230[NH2]
8	A:GLU 175[OE2]	2.63	B:TYR1232[OH]
9	A:ASP 164[OD1]	2.71	B:ARG1234[NE]
10	A:ASP 164[OD2]	2.61	B:ARG1234[NH2]
**Salt bridges**			
**AvrRps4^{C}**		**Dist. [Å]**	**RRS1^{WRKY}**
1	A:GLU 187[OE2]	3.32	B:LYS1221[NZ]
2	A:GLU 175[OE1]	3.50	B:LYS1221[NZ]
3	A:GLU 175[OE2]	3.24	B:ARG1230[NH2]
4	A:ASP 164[OD1]	2.71	B:ARG1234[NE]
5	A:ASP 164[OD2]	3.45	B:ARG1234[NE]
6	A:ASP 164[OD1]	3.41	B:ARG1234[NH2]
7	A:ASP 164[OD2]	2.61	B:ARG1234[NH2]

Table 5. Tukey multiple comparisons of means at 95% family-wise confidence level

Day 3 Col-0

**Construct**	**diff**	**lower bound**	**Upper bound**	**p adj**
D164A-AvrRps4	0.80269085	0.36342384	1.24195786	0.0000053
EE/AA-AvrRps4	1.07949193	0.64022492	1.51875894	0
KRVY/AAAA-AvrRps4	1.14645988	0.70719287	1.58572689	0
N171A-AvrRps4	0.49868711	0.0594201	0.93795413	0.0159246
Q194A-AvrRps4	0.70408364	0.26481663	1.14335065	0.0001012
EE/AA-D164A	0.27680107	-0.1624659	0.71606808	0.4599963
KRVY/AAAA-D164A	0.34376903	-0.095498	0.78303604	0.2192971
N171A-D164A	-0.3040037	-0.7432708	0.13526327	0.3512228
Q194A-D164A	-0.0986072	-0.5378742	0.3406598	0.9873355
KRVY/AAAA-EE/AA	0.06696796	-0.3722991	0.50623497	0.9979272
N171A-EE/AA	-0.5808048	-1.0200718	-0.1415378	0.0025517
Q194A-EE/AA	-0.3754083	-0.8146753	0.06385872	0.1416124
N171A-KRVY/AAAA	-0.6477728	-1.0870398	-0.2085058	0.0004724
Q194A-KRVY/AAAA	-0.4423762	-0.8816433	-0.0031092	0.0472852
Q194A-N171A	0.20539652	-0.2338705	0.64466353	0.759545

Day 3 Ws-2

Construct	diff	Lower bound	Upper bound	p adj
D164A-AvrRps4	1.26186508	0.7822084	1.74152177	0
EE/AA-AvrRps4	1.2783785	0.7987218	1.75803519	0
KRVY/AAAA-AvrRps4	1.12588572	0.646229	1.60554241	0
N171A-AvrRps4	0.492187	0.0125303	0.97184369	0.0405978
Q194A-AvrRps4	0.70761177	0.2279551	1.18726846	0.0004693
EE/AA-D164A	0.01651342	-0.4631433	0.49617011	0.9999987
KRVY/AAAA-D164A	-0.1359794	-0.6156361	0.34367733	0.9644636
N171A-D164A	-0.7696781	-1.2493348	-0.2900214	0.000099
Q194A-D164A	-0.5542533	-1.03391	-0.0745966	0.0132372
KRVY/AAAA-EE/AA	-0.1524928	-0.6321495	0.32716391	0.9425012
N171A-EE/AA	-0.7861915	-1.2658482	-0.3065348	0.0000643
Q194A-EE/AA	-0.5707667	-1.0504234	-0.09111	0.0095929
N171A-KRVY/AAAA	-0.6336987	-1.1133554	-0.154042	0.0025804
Q194A-KRVY/AAAA	-0.418274	-0.8979306	0.06138275	0.1263936
Q194A-N171A	0.21542477	-0.2642319	0.69508146	0.7893133

References

1. L. E. Bird, High throughput construction and small scale expression screening of multi-tag vectors in Escherichia coli. *Methods* **55**, 29-37 (2011).
2. L. Potterton *et al.*, CCP4i2: the new graphical user interface to the CCP4 program suite. *Acta Crystallographica Section D* **74**, 68-84 (2018).
3. A. J. McCoy *et al.*, Phaser crystallographic software. *Journal of Applied Crystallography* **40**, 658-674 (2007).
4. P. Emsley, K. Cowtan, Coot: model-building tools for molecular graphics. *Acta Crystallogr D Biol Crystallogr* **60**, 2126-2132 (2004).
5. T. I. Croll, ISOLDE: a physically realistic environment for model building into low-resolution electron-density maps. *Acta Crystallogr D Struct Biol* **74**, 519-530 (2018).
6. G. N. Murshudov *et al.*, REFMAC5 for the refinement of macromolecular crystal structures. *Acta Crystallographica Section D* **67**, 355-367 (2011).
7. C. J. Williams *et al.*, MolProbity: More and better reference data for improved all-atom structure validation. *Protein Sci* **27**, 293-315 (2018).
8. G. Battle (2016) PDBePISA : Identifying and interpreting the likely biological assemblies of a protein structure.
9. E. F. Pettersen *et al.*, UCSF ChimeraX: Structure visualization for researchers, educators, and developers. *Protein Sci* **30**, 70-82 (2021).
10. H. Wickham, ggplot2-Elegant Graphics for Data Analysis. Springer International Publishing. *Cham, Switzerland* (2016).
11. Á. Piñeiro *et al.*, AFFINImeter: A software to analyze molecular recognition processes from experimental data. *Analytical Biochemistry* **577**, 117-134 (2019).
12. Panagiotis F. Sarris *et al.*, A Plant Immune Receptor Detects Pathogen Effectors that Target WRKY Transcription Factors. *Cell* **161**, 1089-1100 (2015).
13. Y. Ma *et al.*, Distinct modes of derepression of an Arabidopsis immune receptor complex by two different bacterial effectors. *Proceedings of the National Academy of Sciences* **115**, 10218 (2018).
14. K. H. Sohn, R. K. Hughes, S. J. Piquerez, J. D. G. Jones, M. J. Banfield, Distinct regions of the Pseudomonas syringae coiled-coil effector AvrRps4 are required for activation of immunity. *Proceedings of the National Academy of Sciences* **109**, 16371 (2012).
15. C. E. M. Stevenson, D. M. Lawson, Analysis of Protein-DNA Interactions Using Surface Plasmon Resonance and a ReDCaT Chip. *Methods Mol Biol* **2263**, 369-379 (2021).
16. H. Ashkenazy *et al.*, ConSurf 2016: an improved methodology to estimate and visualize evolutionary conservation in macromolecules. *Nucleic Acids Research* **44**, W344-W350 (2016).
